# From Sampling to Sequencing: A Liquid Biopsy Pre-Analytic Workflow to Maximize Multi-Layer Genomic Information from a Single Tube

**DOI:** 10.3390/cancers13123002

**Published:** 2021-06-15

**Authors:** Kendra K. Maass, Paulina S. Schad, Agnes M. E. Finster, Pitithat Puranachot, Fabian Rosing, Tatjana Wedig, Nathalie Schwarz, Natalie Stumpf, Stefan M. Pfister, Kristian W. Pajtler

**Affiliations:** 1 German Cancer Research Center (DKFZ), Division of Pediatric Neurooncology, Hopp-Children’s Cancer Center Heidelberg (KiTZ) and German Cancer Consortium (DKTK), 69120 Heidelberg, Germany; p.schad@kitz-heidelberg.de (P.S.S.); a.finster@kitz-heidelberg.de (A.M.E.F.); f.rosing@kitz-heidelberg.de (F.R.); t.wedig@kitz-heidelberg.de (T.W.); n.schwarz@kitz-heidelberg.de (N.S.); n.stumpf@dkfz-heidelberg.de (N.S.); s.pfister@kitz-heidelberg.de (S.M.P.); 2 Department of Pediatric Hematology and Oncology, Heidelberg University Hospital, 69120 Heidelberg, Germany; 3 Division of Applied Bioinformatics, German Cancer Research Center (DKFZ), 69120 Heidelberg, Germany; p.puranachot@dkfz-heidelberg.de; 4 Faculty of Medicine and Public Health, HRH Princess Chulabhorn College of Medical Science, Chulabhorn Royal Academy, Bangkok 10210, Thailand

**Keywords:** liquid biopsy, cell-free DNA, cell-free RNA, blood preservation tubes, CSF, size selection, droplet digital PCR, low-coverage whole-genome sequencing, cancer, pre-analytics

## Abstract

**Simple Summary:**

Liquid biopsies seek to isolate tumor derived genetic material that circulates in blood plasma or cerebrospinal fluid. The less-invasive character of liquid biopsies combined with the option for serial analyses bears enormous potential for treatment monitoring or surveillance. We aimed to establish robust sampling protocols and pre-analytical workflows to allow for site independent multi-layer liquid biopsy testing. For an optimal usage of precious material, we explored sample stabilization in various conservation tubes and describe a protocol for the parallel isolation of cell-free DNA and RNA. Quantification and quality control steps were optimized for minimal sample use with both high sensitivity and reproducibility. We provide detailed step-by-step information on how to i) choose the best-suited protocol and ii) implement this in the liquid biopsy workflow. We believe that our study has potential to increase comparability of liquid biopsy approaches to bring these one step closer to routine clinical application.

**Abstract:**

Liquid biopsies hold great promise for the management of cancer. Reliable liquid biopsy data depend on stable and reproducible pre-analytical protocols that comply with quality measures, irrespective of the sampling and processing site. We established a workflow for plasma preservation, followed by processing, cell-free nucleic acid isolation, quantification, and enrichment of potentially tumor-derived cell-free DNA and RNA. Employing the same input material for a direct comparison of different kits and protocols allowed us to formulate unbiased recommendations for sample collection, storage, and processing. The presented workflow integrates the stabilization in Norgen, PAX, or Streck tubes and subsequent parallel isolation of cell-free DNA and RNA with NucleoSnap and NucleoSpin. Qubit, Bioanalyzer, and TapeStation quantification and quality control steps were optimized for minimal sample use and high sensitivity and reproducibility. We show the efficiency of the proposed workflow by successful droplet digital PCR amplification of both cell-free DNA and RNA and by detection of tumor-specific alterations in low-coverage whole-genome sequencing and DNA methylation profiling of plasma-derived cell-free DNA. For the first time, we demonstrated successful parallel extraction of cell-free DNA and RNA from plasma samples. This workflow paves the road towards multi-layer genomic analysis from one single liquid biopsy sample.

## 1. Introduction

The term liquid biopsy comprises the broad spectrum of possibilities arising from the detection and analysis of different potential biomarkers in body fluids, with blood plasma being the most prominent example. Possible candidates range from circulating tumor cells, tumor-derived nucleic acids, circulating extracellular vesicles, metabolites, to proteins [1]. Liquid biopsies have shown promising results as a non-invasive, complementary diagnostic tool in the management of cancer [2,3,4,5,6]. Due to their minimally invasive nature, liquid biopsies provide certain advantages over traditional tumor biopsies. For example, they can be collected serially over the course of treatment, they may be collected by a general practitioner, and they might represent all tumor sites in the body [7,8]. In addition, many studies in this field are evaluating early cancer detection with broad screening approaches and long-term surveillance of cancer survivors with liquid biopsies [9,10,11,12,13]. Prerequisite for all above-mentioned liquid biopsy applications are robust protocols for sampling and pre-analytical workflows which are considered in this study.

With the molecular landscape of many cancer entities having been deciphered, in research studies, cell-free DNA (cfDNA) in particular and to a lower extent cell-free RNA (cfRNA) have predominantly been chosen as circulating analytes to follow response to treatment in order to identify early therapeutic resistance and to adjust targeted therapies accordingly [14,15,16]. As tumors display increased proliferation rates, their cells contribute comparatively high amounts to the total cfDNA and cfRNA pool in the circulation. The main source of cell-free nucleic acid (cfNA) release is most likely a passive process via apoptosis and necrosis [17,18,19]. Depending on tumor size, location, vascularization, stage, and the presence of physiological barriers, such as the blood–brain barrier, elevated levels of circulating tumor DNA (ctDNA) or RNA (ctRNA) can be detected at variable fractions in the cfNA, ranging between 0.01% and 90% [20].

One of the main factors for successful tumor detection through liquid biopsies is the pre-analytical workflow. In this context, prevention of leukocyte lysis, the major source of normal genomic DNA (gDNA) contamination, is essential, as excessive gDNA concentrations can interfere with successful cfDNA analysis [21,22]. Among other reasons, this is why methodologies to analyze cfDNA in biological fluids are usually technologically more demanding than those of tissue analyses. Leukocyte lysis can be prevented through adapted blood collection protocols. This entails the use of blood preservation tubes in combination with detailed handling manuals, optimized transportation, and thorough sample processing.

Another possibility to improve liquid biopsy sequencing results lies in the non-random fragmentation patterns of cfDNA in human plasma. DNA associated with nucleosomes is better protected from enzymatic processes such as apoptotic caspases in the circulation [23,24,25]. This results in characteristic cfDNA peaks of around 166 base pairs (bp) comprising DNA wrapped around one nucleosome (≈146 bp) together with linker DNA (≈20 bp) or multiples thereof (160–500 bp) [26,27]. In cancer patients, shorter fragments in particular were determined to harbor tumor-specific mutations [28]. These size characteristics of cfDNA can be leveraged for pre-analytical enrichment of ctDNA by physical size selection of the cfDNA pool.

Limitations identified in several liquid biopsy studies based on cfDNA analysis included a plateau in sensitivity and specificity for the detection of localized cancers regardless of the analysis method [10]. This could potentially be improved by the combination of different biomarkers, such as cfDNA and proteins [11] or cfDNA and cfRNA. The possible advantages of combining the analysis of different biomarkers from one tube to obtain a broader picture of the tumor has not yet been sufficiently characterized for liquid biopsy diagnostics.

Stable and reproducible pre-analytical and analytical protocols are necessary in order to address these technical challenges associated with liquid biopsy analysis. In this study, we established workflows that allow multi-layer liquid biopsy testing and indicate important pre-analytical considerations. We provide an overview of results obtained from different blood preservation tubes, cfNA isolation kits, different quantification methods for low concentration ranges, and a size selection protocol to enrich for ctDNA. Finally, we investigated the yield and integrity of cfDNA and cfRNA by droplet digital PCR (ddPCR), low-coverage whole-genome sequencing (lcWGS), and DNA methylation profiling to illustrate the efficacy of the optimized workflow from sampling to sequencing. Especially in pediatric oncology, but also in critically ill cancer patients, plasma volumes are limited and usually do not allow separate isolation procedures for the different biomarkers.

To our knowledge, this is the first study to describe protocols for the successful co-analysis of cfDNA and cfRNA. Herein, we discuss the potential and limitations of combined cfNA analysis. We provide information on how to choose the best-suited protocol and how to implement it in the liquid biopsy workflow. By making this pipeline available, we hope to contribute to more consistent compliance irrespective of the sampling and processing site and to bring liquid biopsies one step closer to routine clinical practice.

## 2. Results

### 2.1. Impact of Liquid Biopsy Collection Tubes on Plasma cfDNA Yield and Purity

**Figure 1:**
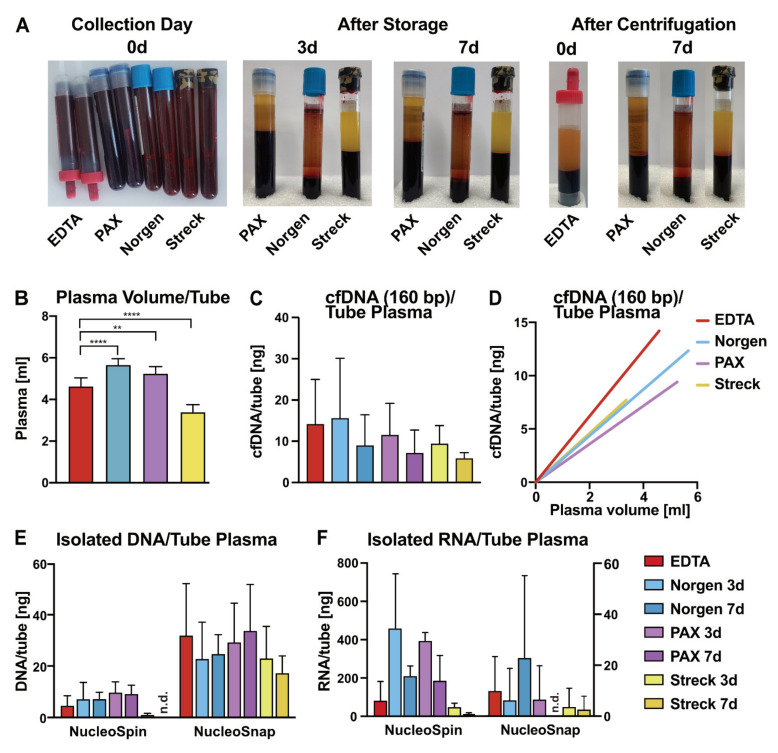
Comparative analysis of blood conservation tubes. (**A**) Representative images of blood collected in EDTA, PAX, Norgen, and Streck tubes at the same time point from the same healthy donor for a direct comparison of tube performance. Pictures were taken right after blood drawing (left), after 3 d (middle left), after 7 d (middle right), and after plasma processing by centrifugation (right). (**B**) Mean plasma volumes obtained from indicated blood conservation tubes after centrifugation (*n* = 4). ** *p* < 0.01, **** *p* < 0.0001 as determined by Kruskal–Wallis test (**C**) The mean concentration of cfDNA recovered from indicated tubes isolated by NucleoSnap quantified on Bioanalyzer profile (BA). The size of DNA wrapped around one nucleosome can range from around 146 to 176 bp and is represented by 160 bp (*n* = 4). (**D**) cfDNA yield per plasma volume calculated for the different tubes. Concentration is defined as cfDNA (160 bp, measured by BA) per plasma volume (*n* = 4). (**E**,**F**) Mean Qubit concentration of total DNA (**E**) and total RNA (**F**) recovered from indicated tubes isolated by NucleoSpin (left) or NucleoSnap (right). n.d. indicates not detectable values. When no specific time point is indicated, values represent the mean of both time points (*n* = 4).

When full blood cannot be centrifuged directly after blood collection or when necessary equipment is not available at the collection site, blood preservation tubes can be considered to improve plasma quality through reduced lysis of leukocytes. We tested K_3_EDTA S-monovettes (EDTA) tubes against three different commercially available blood preservation tubes for cfNA yield and purity. For each healthy person (P1–P4, *n* = 4; Table S1), blood was collected into two EDTA tubes and into two of each cf-DNA/cf-RNA preservative tubes, namely, cf-DNA/cf-RNA Preservative Tubes Norgen (Norgen), PAXgene Blood ccfDNA Tube IVD (PAX), and Cell-Free DNA BCT Streck (Streck) tubes (Table S1). EDTA tubes were processed to plasma within one hour after blood collection. The other tubes were stored for 3 and 7 d at room temperature (RT). The collection tubes were evaluated for visual characteristics right after blood collection, after 3 and 7 d incubation times, and after plasma generation through centrifugation (Figure 1A). Significant differences in the plasma volumes obtained were observed between EDTA (mean = 4.59 mL) and Norgen (mean = 5.67 mL, *p* < 0.0001) or PAX (mean = 5.26 mL, *p* = 0.0021), with all three yielding more plasma than Streck (mean = 3.48 mL, *p* < 0.0001; Figure 1A,B). This may have been due to the fact that the companies use different preservation chemistries resulting in different dilutions of the plasma. According to manufacturers, Norgen tubes rely on osmotic stabilization of nucleated cells, PAX tubes employ biological apoptosis inhibition, and Streck uses a chemical crosslinking approach (Table 1).

For further analysis, cfDNA was defined in the range from 146 to 176 bp (i.e., length of DNA wrapped around one nucleosome) and is represented by the 160 bp peak (Figure S2A). The yields of cfDNA were measured by Bioanalyzer DNA HS (BA) and revealed the highest amounts in Norgen followed by EDTA, PAX, and Streck tubes (Figure 1C). The performance of the different tubes was expressed as cfDNA purity, which was defined as the ratio of cfDNA (160 bp, Figure S2A) to total DNA (50–7000 bp, Figure S2C), thereby quantifying the genomic contamination. For the most accurate analysis possible, we, in our study, defined cfDNA as DNA wrapped around one nucleosome only. Broader definitions of cfDNA, including DNA wrapped around one to three nucleosomes with fragment sizes of up to 500 bp (Figure S2B), are, however, also possible. How different definitions impact measured cfDNA amounts is illustrated in Figure S2D. cfDNA purity values were similar between all tested tubes and were all approaching a purity ratio of 1, indicative of successful prevention of leukocyte lysis (Figure S2E). While the largest plasma volumes were recovered from Norgen and PAX tubes, cfDNA isolated with NucleoSnap cfDNA kit (NucleoSnap) per plasma volume was observed to be the same or slightly lower compared to others (Figure 1D). To compare the total amounts of cfNA that can be isolated from EDTA, Norgen, and PAX tubes, we divided plasma volumes into half and subjected them to co-isolation of cfDNA and cfRNA by either NucleoSnap, designed for cfDNA isolation, or NucleoSpin miRNA Plasma kit (NucleoSpin), designed for cfRNA isolation. Both isolation kits allowed the co-isolation of cfDNA and cfRNA in varying ratios. The lower plasma volumes in Streck tubes did not allow for the separation into two fractions. Therefore, two separate tubes were used for the isolation with NucleoSnap and NucleoSpin. Considering the amounts of cfDNA isolated per tube, all tubes achieved higher results for total isolated cfDNA when using NucleoSnap instead of NucleoSpin (Figure 1E, Figures S1A and S2F–I). Over the course of 3 to 7 d, a slight decrease of cfDNA amounts was observed (Figure 1C and Figure S2F–I), while the total DNA amount increased in all tubes except Streck, potentially caused by contamination with gDNA (Figure 1E). This was most likely due to increasing leukocyte lysis rates over time and it is therefore recommended that the plasma is processed at the earliest time point possible for optimal plasma quality and cfNA isolation. With both NucleoSnap and NucleoSpin, cfRNA was barely detectable in Streck tubes, while reasonable yields were isolated from EDTA, Norgen, and PAX tubes (Figure 1F). With the exception of Norgen tubes isolated with NucleoSnap, cfRNA yields revealed a pronounced decrease over time reinforcing the earliest possible plasma generation due to RNA instability (Figure 1F). Combined analysis of RNA measurements from NucleoSpin vs. NucleoSnap revealed a significantly higher RNA yield for NucleoSpin (Figure S1B). When we combined cfDNA and cfRNA results over all tubes, NucleoSnap appeared to allow for the broadest spectrum of cfNA analysis.

### 2.2. Impact of Liquid Biopsy Collection Tubes on Cerebrospinal Fluid cfDNA Yield and Purity

**Figure 2:**
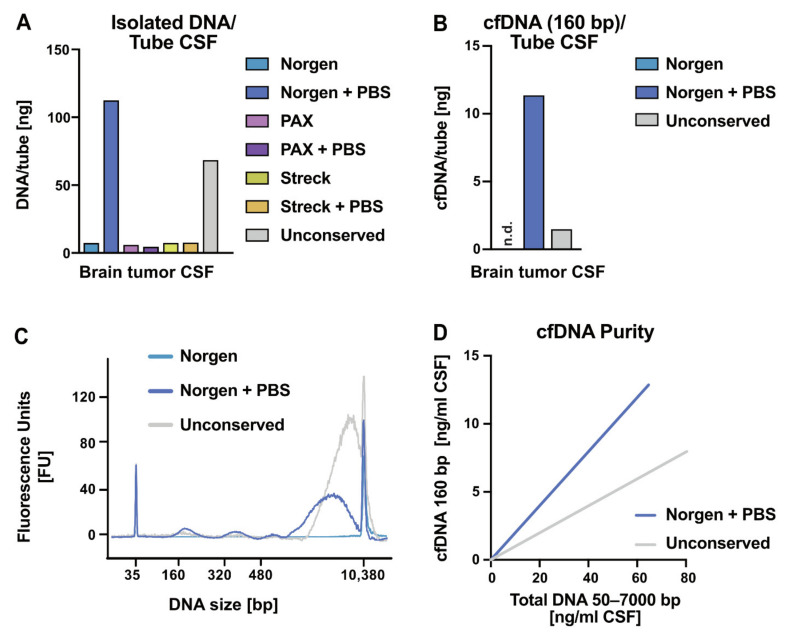
Strategies for CSF conservation in blood preservation tubes. (**A**) Concentration of total DNA recovered from 1.5 mL CSF of a brain tumor patient conserved in the indicated tube type. DNA was isolated with NucleoSpin, and the DNA concentration was measured by Qubit. (**B**) The concentration of cfDNA (first nucleosomal peak, 160 bp) recovered from 1.5 mL CSF of a brain tumor patient conserved in the indicated tube for preservation and isolated by NucleoSpin. The concentration was determined by measuring the first peak of the Bioanalyzer profile (BA). n.d. indicates not detectable values. (**C**) BA profiles for DNA isolated from CSF of a brain tumor patient after 7 d unconserved (gray), in a Norgen tube (light blue), and a Norgen tube filled up with PBS before centrifugation (dark blue). (**D**) cfDNA purity of DNA isolations from unconserved CSF and CSF collected in a Norgen tube that was filled up with PBS. Purity was defined as the ratio of cfDNA (first nucleosomal peak, 160 bp) to total DNA (50–7000 bp) measured by BA.

Several studies have suggested cerebrospinal fluid (CSF) as a superior source over blood plasma for successful liquid biopsy detection of brain tumors [29,30,31]. However, studies on pre-analytical considerations for CSF are sparse. We aimed to test CSF conservation by simulating clinical conditions. This included comparably small volumes typically obtained from a lumbar puncture, no direct use of preservation tube adaptors—hence tube filling by breaking the preservation tubes’ vacuum—and conservation in a ratio of CSF to preservation reagent of around 1:1. We compared the same preservation tubes as we did for the blood samples. Irrespective of tube conservation, CSF of a non-cancerous person revealed only marginal concentrations of total DNA, but no cfDNA peak on the BA (Figure S3A,B, Table S1). Moreover, we added 1.5 mL aliquots of CSF from a brain tumor patient into two tubes each for Norgen, PAX, and Streck, and stored them for 7 d at RT. One additional aliquot served as a control without addition of any conservative agent and was stored for 7 d at 4 °C in an Eppendorf tube. Norgen recommends to fill up incompletely filled tubes with phosphate-buffered saline (PBS) before centrifugation. We decided to evaluate this factor for all different tubes. The amount of total DNA isolated with NucleoSpin from the different tubes centrifuged with and without the addition of PBS confirmed the beneficial effect of PBS addition to Norgen tubes, but not the others (Figure 2A). We applied NucleoSpin for parallel extraction of cfDNA and cfRNA. However, cfRNA isolation did not lead to measurable amounts of cfRNA (data not shown). The detection of distinct cfDNA peaks in the BA profile was only possible from CSF samples without conservation (unconserved) and from Norgen tubes filled up with PBS right before centrifugation (Figure 2B,C). The direct comparison of BA profiles of unconserved CSF and Norgen tube with and without PBS showed that the use of a preservation tube in combination with PBS filling was superior to all other tested conditions by stabilizing the cfDNA together with less gDNA contamination (Figure 2C). Of note, the Norgen tube profiles that were processed without PBS did not result in a detectable cfDNA peak on the BA profile, emphasizing the importance of PBS addition (Figure 2B,C). Using ddPCR as a more sensitive detection method, we were able to observe an appreciable increase in all tubes for cfDNA and cfRNA after the addition of PBS before centrifugation (Figure S3C–E). The purity was quantified as cfDNA (160 bp) yields against total DNA (50–7000 bp), revealing the highest purity for Norgen with PBS followed by the unconserved sample (Figure 2D). With no cfDNA peak detected in the BA profile of Norgen without PBS addition, we could not assess and visualize the purity of Norgen without PBS (Figure 2D). Due to the limited access to CSF in general, this experimental section has limitations in the cohort size and only allows for preliminary conclusions.

### 2.3. Comparative Analysis of Different cfNA Isolation Kits

**Figure 3:**
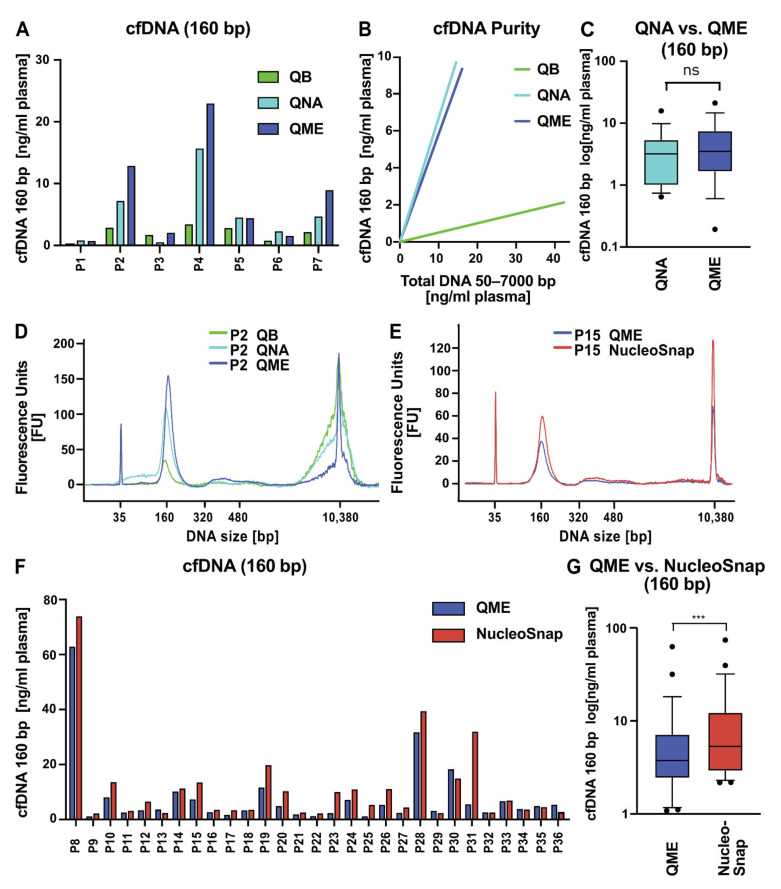
Comparative evaluation of cell-free DNA isolation kit performance concerning yield and purity. (**A**) Direct, paired comparison of QIAamp DNA Blood Mini Kit (QB, green), QIAamp Circulating Nucleic Acid Kit (QNA, light blue), and QIAamp MinElute ccfDNA Mini Kit (QME, dark blue) isolation kits on plasma aliquots of pediatric cancer patients (*n* = 7). Cell-free DNA (cfDNA) yields are depicted as the concentration of cfDNA in the first nucleosome peak (160 bp) measured by Bioanalyzer (BA) per millilter of plasma. (**B**) cfDNA purity was defined as the ratio of cfDNA (first nucleosomal peak, 160 bp) to total DNA (50–7000 bp) measured by BA. QB= 0.05; QNA= 0.668; QME= 0.582. (**C**) Plasma aliquots of pediatric cancer patients were isolated with QNA and QME for direct comparison. Logarithmic representation of cfDNA yields measured as first nucleosomal peak (160 bp) by BA. Boxplot shows median and interquartile range, whiskers represent 10th–90th percentiles (*n* = 16, *p* = 0.13). ns denotes differences in means that were not significant as determined by Wilcoxon signed-rank test. (**D**) Representative BA profiles of one plasma sample (P2 cf. Figure 3A) isolated with QB (green), QNA (light blue), and QME (dark blue). (**E**) Representative BA profiles of one plasma sample (P15 cf. Figure 3F) isolated with QME (dark blue) and NucleoSnap (red). (**F**,**G**) Direct comparison of QME and NucleoSnap isolation kit performance for plasma aliquots of pediatric cancer patients. (**F**) cfDNA yields measured with BA are depicted as the concentration of cfDNA (first nucleosomal peak, 160 bp) per milliliter of plasma. (**G**) Logarithmic representation of cfDNA yields (first nucleosomal peak, 160 bp) by BA. Boxplot shows median and interquartile range, whiskers represent 10th–90th percentiles (*n* = 29, *p* = 0.0005). *** *p* < 0.001 as determined by Wilcoxon signed-rank test.

The next important step after successful plasma generation is the efficient isolation of cfDNA. Five different nucleic acid isolation kits were evaluated for cfDNA yield and purity. As a first step, we divided EDTA-derived plasma from seven patients into three aliquots for direct comparison of QIAamp DNA Blood Mini Kit (QB), QIAamp Circulating Nucleic Acid Kit (QNA), and QIAamp MinElute ccfDNA Mini Kit (QME) (Table S1). For comparability, the amount of cfDNA was standardized to the plasma input volume. The quantification of cfDNA isolated with the different kits and analyzed with BA revealed a superiority of QNA and QME (Figure 3A). When looking at the purity by analyzing the ratio of cfDNA (160 bp) to total DNA (50–7000 bp), we found that QNA and QME also yielded considerably purer cfDNA samples than QB (Figure 3B). QB had the highest amount of total DNA but with only a minor fraction of cfDNA and was therefore not included in the more thorough comparison (Figure S4A–C). We included another nine EDTA-derived plasma samples for direct comparison of QNA and QME (total *n* = 16, Table S1) and observed slightly higher amounts of cfDNA for QME but no significant difference (Figure 3C, *p* = 0.13; Figure S4D). Figure 3D shows representative BA profiles of plasma from patient 2 (P2, Figure 3A) after isolation with the three different kits. QNA and QME had higher cfDNA yields than QB, with QME showing the lowest gDNA contamination.

Having established QME as the most efficient cfDNA isolation kit among the three kits tested, we then went on to compare QME to NucleoSnap using EDTA-derived plasma aliquots from 29 patients, standardized to the plasma volume (Table S1). NucleoSnap consistently achieved significantly higher cfDNA yields over almost all plasma samples tested (22/29) (*n* = 29, *p* = 0.0005; Figure 3F,G). The exemplary BA DNA size profile overlay of patient 14 (P14; Figure 3E) illustrated clear cfDNA peaks with low degrees of gDNA contamination for both QME and NucleoSnap, but with higher amounts of cfDNA and similar levels of gDNA contamination for NucleoSnap. Regarding cfDNA purity in general, NucleoSnap showed a slightly worse performance than QME (Figure S4E). In an optimal pre-analytical setting with timely plasma generation or the use of cfDNA stabiliziation tubes, the occurrence of gDNA contamination is usually not as frequent as it was the case in some of our patient samples. For cases such as these, the removal of larger fragments by size selection can compensate for the lower purity, but NucleoSnap still recovered the highest cfDNA yields. These results point to NucleoSnap as the superior method for cfNA isolation outperforming QB, QNA, QME (Figure 3), and NucleoSpin (Figure 1).

### 2.4. Evaluation of DNA Quantification Methods for Low-Concentration Samples

**Figure 4:**
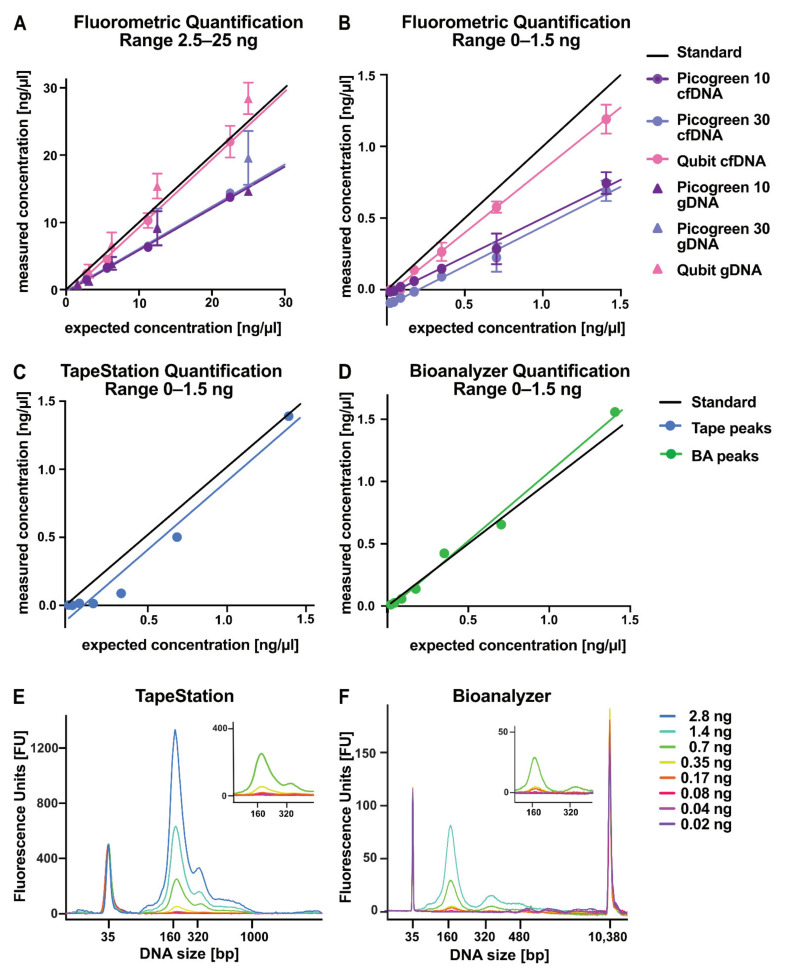
Evaluation of sensitivity and reproducibility of DNA quantification methods in low concentration range. (**A**,**B**) Correlation of expected to measured concentration of genomic DNA (gDNA) and cell-free DNA (cfDNA) quantified by fluorometric measurement methods (*n* = 5). (**A**) gDNA and cfDNA concentration in the range of 2.5–25 ng/µL. (**B**) The range of 0–1.5 ng/µL is indicated only for cfDNA values. (**C**,**D**) Quantification of cfDNA by electrophoretic quantification methods. (**C**) cfDNA of indicated concentrations were run on TapeStation cfDNA screen tapes and quantified for the peak region. (**D**) cfDNA of indicated concentrations were run on Bioanalyzer (BA) chip and the observed peaks were quantified. (**E**) Overlay of corresponding TapeStation cfDNA profiles of indicated concentration (0.02–2.8 ng) quantified in (**C**). In the upper right corner: zoom-in of the TapeStation profiles ≤ 700 pg. (**F**) Overlay of corresponding BA cfDNA profiles of indicated concentrations (0.0–21.4 ng) quantified in (**D**). In the upper right corner: zoom-in of the BA profiles ≤ 700 pg. All whiskers represent the standard deviation of technical replicates.

Liquid biopsy analyses are often hampered by minimal amounts of isolated analytes. After cfDNA isolation, the next critical steps are a sensitive and robust quantification and thorough quality control. Therefore, four quantification methods were compared in terms of sensitivity and reproducibility. For fluorometric quantification methods, we compared Qubit DNA HS assay to the PicoGreen DNA HS assay, performing the latter with two different standard curves (PicoGreen 10/PicoGreen 30; Figure 4A,B). We included gDNA (triangle) and cfDNA (circle) dilution series ranging from 2.5 to 25 ng/µL to test whether DNA type influenced the assay performance. PicoGreen underestimated the actual input amounts, while Qubit was reproducibly closer to the expected DNA concentrations (Figure 4A). We then took a closer look at the assay performance and reproducibility for cfDNA in the low concentration range of 0–1.5 ng/µL (Figure 4B). We observed a similar trend with PicoGreen underestimating the DNA concentration, which even resulted in negative values for concentrations lower than 0.17 ng/µL. In addition to fluorometric assays to assess total concentration, electrophoretic assays, such as BA DNA HS chip and TapeStation cell-free DNA tape, allowed for both quantification and quality control by size separation of the isolation product. The cell-free DNA ScreenTape for TapeStation Systems is specifically designed for the analysis of cfDNA samples with a range from 50 to 1000 bp length (Figure 4C,E). Both TapeStation and BA assays resulted in measured concentrations consistent with expected concentrations, particularly within the range specified by the manufacturer (TapeStation 20–4000 pg/µL, BA 5–1000 pg/µL) (Figure 4C,D). The electrophoretic profiles of both methods revealed clear cfDNA peaks with slightly different characteristics. The cell-free DNA ScreenTape selectively showed DNA in the size range of 0–1000 bp (Figure 4E). In contrast, the BA DNA HS Chip depicts a much broader range from 0–10,380 bp and therefore allows for a clear separation of cfDNA and gDNA (Figure 4F). From the quantification methods evaluated here, Qubit, BA, and TapeStation showed the best results in our analyses.

### 2.5. Enrichment of cfDNA by Size Selection

**Figure 5:**
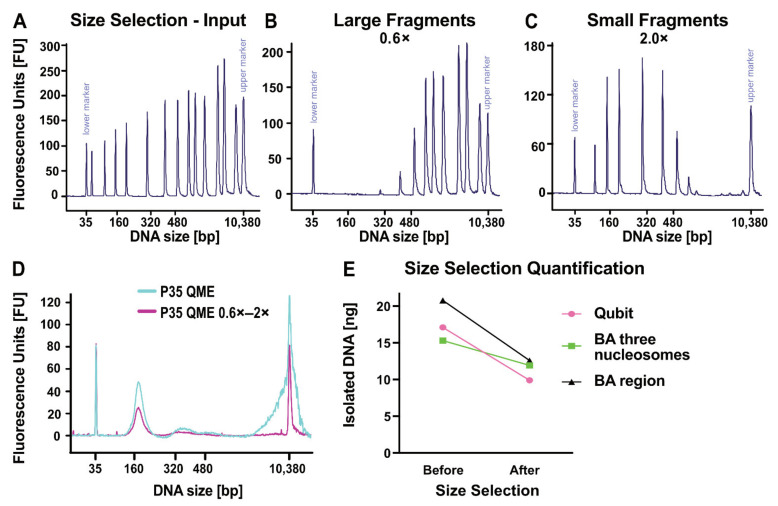
Size selection strategies for specific enrichment of cell-free DNA. (**A**) Bioanalyzer (BA) profile of DNA HS ladder with defined peak sizes serving as input for size selection. (**B**) DNA ladder from (A) after performing right side size selection. 0.6× bead bound eluate loaded. (**C**) DNA ladder from (A), with small DNA being in the 0.6× supernatant. Small fragments were bound in a second step by a 2.0× bead ratio and subsequently eluted. (**D**) cfDNA of P35 isolated with QIAamp MinElute ccfDNA Mini Kit (QME) was subjected to size selection. DNA profile before (light blue) and after size selection (pink) employing the 0.6x bead ratio to separate cfDNA from larger DNA fragments. (**E**) Quantification of total DNA (pink) by Qubit measurement and of cfDNA peaks (160–480 bp, light green) and total DNA (region 50–7000 bp, black) by BA measurement before and after size selection.

Physical separation of cfDNA from gDNA according to size might be necessary for certain downstream analyses, such as ddPCR or methylation arrays, where the method itself does not discriminate between short and long DNA fragments. In contrast to this, library preparation processes preferably incorporate shorter fragments due to the specifics of the PCR amplification step, and therefore no prior size selection is required. To find the best-suited size selection ratio for retaining potential ctDNA fragments while removing most gDNA contaminants, we subjected a DNA ladder of the BA DNA HS Assay with well-defined peak sizes to magnetic bead size selection in varying ratios (Figure S5A–J). The basic principle of the size selection process was visualized with the input ladder (Figure 5A), the removed larger fragments bound at 0.6× bead ratio (Figure 5B)—mimicking possible gDNA contamination—and the supernatant fraction with shorter DNA fragments (Figure 5C). In detail, first the longer gDNA fragments were bound to beads in a ratio of 0.6× (Figure 5B). Subsequently, the resulting supernatant was subjected to a final bead ratio of 2.0×, thereby inducing the binding of shorter cfDNA fragments to the beads (Figure 5C). On the basis of this, we chose a bead ratio of 0.6× to eliminate gDNA contamination while keeping cfDNA peaks of one, two, and three nucleosomal peaks with a size of up to 500 bp (Figure 5A–C and Figure S2B). DNA isolated from plasma of patient P35 was subjected to size selection because of a high proportion of large gDNA fragments and analyzed before and afterwards by BA. Respective BA profiles indicate the separation of gDNA after size selection with retention of the cfDNA peak (Figure 5D). This was illustrated by Qubit and BA quantification measurements where, we observed a decrease in the total amount of DNA as determined by Qubit and BA region (50–7000 bp) measurement. For cfDNA (three nucleosomal peaks, 160–500 bp), we observed a total decrease of 22%, which resulted in an increased cfDNA purity of 95% compared to 81% before size selection (Figure 5E).

### 2.6. Performance of cfNA in Downstream Analyses

**Figure 6:**
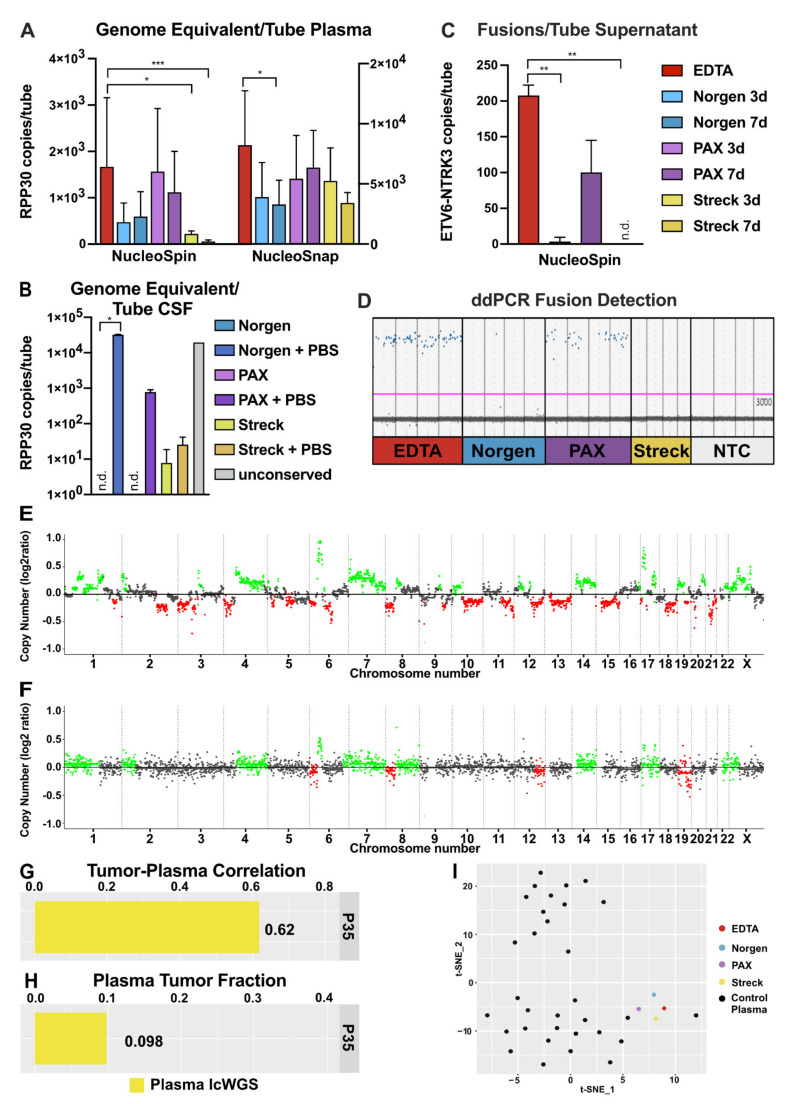
Cell-free nucleic acid validation after application of the improved pre-analytical protocol. (**A**) Genome equivalents represented as copies of the non-amplified genome region RPP30 per indicated plasma tube plotted for NucleoSpin (left) and NucleoSnap (right). Error bars indicate standard deviation (*n* = 4). ** *p* < 0.01, *** *p* < 0.001 as determined by Kruskal–Wallis test. (**B**) Genome equivalents from CSF from indicated tubes represented as copies of the non-amplified genome region RPP30 plotted as copies per tube. Error bars are plotted as a standard deviation from technical replicates (*n* = 2). n.d. indicates not detectable values. * *p* < 0.05 as determined by Kruskal–Wallis test. (**C**) Transcriptome equivalents from cell culture supernatant represented as ETV6-NTRK3-fusion transcripts as copies per tube (*n* = 4). n.d. indicates not detectable values. ** *p* < 0.01 as determined by Kruskal–Wallis test. (**D**) ddPCR data depicting either fusion-positive (blue) or fusion-negative (gray) droplets in the different tubes. (**E**) Copy number variation (CNV) profile of osteosarcoma tumor DNA obtained from methylation EPIC array and (**F**) corresponding low-coverage whole-genome sequencing (lcWGS) of plasma collected from patient P35 following application of an optimized cell-free DNA (cfDNA) isolation protocol. (**G**) Correlation coefficient of the tumor CNV profile and the matching plasma CNV profile from lcWGS (yellow). (**H**) Estimated tumor fraction representing the relative amount of circulating tumor DNA (ctDNA) in the overall cell-free DNA (cfDNA) pool. (**I**) cfDNA from one individual stored in different conservation tubes clustered in close proximity when subjected to methylation analysis. T-SNE approach was used for reduction of dimensionality.

The final step of the pre-analytic workflow was to test the cfNA stability and the performance of the final cfNA products resulting from the above-described workflow in downstream sequencing techniques. With ddPCR, we assessed the integrity and abundance of genome equivalents represented by copies of the non-amplified genome region RPP30 detected per plasma tube (Figure 6A). The highest numbers of RPP30 copies were detected in EDTA and PAX tubes, followed by Norgen and Streck. For NucleoSpin analysis, Streck yielded significantly lower values than EDTA (*p* = 0.0332 and *p* = 0.0002), while for NucleoSnap, a significant difference was observed between EDTA and Norgen at 7 d (*p* = 0.0364). When performing the ddPCR assays on cfDNA isolated from CSF (Figure 2 and Figure S3), we observed a higher detection rate by RPP30 ddPCR compared to the quantification methods described above (Figure 6B). Despite low DNA concentrations detected by Qubit measurements for CSF in Norgen (*p* = 0.0385) and PAX tubes without the addition of PBS, the ddPCR assay for RPP30 could not detect amplifiable DNA, while similar DNA concentrations resulted in positive counts for Streck with and without the addition of PBS (Figure 6B). This emphasized the importance of validating the integrity of isolated DNA not only by quantification and size profiling but also with integrity assays.

RNA integrity was assessed by quantifying ETV6-NTRK3 fusion transcripts (Figure 6C,D). Fusion transcripts were detectable in cell culture supernatant collected into EDTA or preserved in PAX tubes, while almost nothing (Norgen; *p* = 0.0097) or nothing at all was detectable in the other tubes (Streck; *p* = 0.0034). In addition, we assessed the GAPDH mRNA copies as transcript equivalents in the preserved plasma after isolation with NucleoSnap. Comparable to genome equivalents, most copies were detected in EDTA and PAX tubes followed by Norgen (Figure S6). To assess the quality of the final cfDNA product in downstream sequencing analysis, we performed lcWGS of cfDNA from patient P35 (Figure 3F and Figure 5D) isolated with the proposed pre-analytical workflow. We compared the resulting copy number variation (CNV) profile of the cfDNA to the respective tumor profile obtained from a tissue biopsy (Figure 6E,F). We observed tumor-specific chromosomal gains and losses in the lcWGS CNV plot (Figure 6F), a correlation of 60% between tumor tissue and plasma CNV profiles (Figure 6G), and a tumor fraction of 0.1 in the cfDNA (Figure 6H). The impact of the different collection tubes on the methylation patterns in cfDNA was investigated by methylation EPIC array. T-distributed stochastic neighbor embedding (tSNE) clustering revealed that all cfDNA isolates from one individual preserved for 7 days in the different tubes clustered in close proximity to the EDTA sample (Figure 6I). This suggests that all tubes are compatible with subsequent DNA methylation profiling.

## 3. Discussion

Pre-analytical factors can significantly affect liquid biopsy analyses. While many studies focus on specific aspects of the workflow, pre-analytical guidelines from start to finish including the combined analysis of cfDNA and cfRNA are missing. We have established an upfront pre-analytical pipeline including the choice of preservation tubes, sample centrifugation, efficient co-isolation of cfDNA and cfRNA, quantification, and quality assessment. Co-isolation of cfRNA can add a complementary layer of information and thereby increase the sensitivity and specificity of the liquid biopsy analysis. The multi-marker protocols developed in this manuscript can help to design well-prepared liquid biopsy studies with precious body fluid material to achieve a reproducible performance of downstream analyses.

### 3.1. Liquid Biopsy Preservation Tubes

Delays between blood collection and cfDNA isolation can lead to higher degrees of gDNA and cellular RNA contamination through leukocyte lysis, thus hampering downstream analyses [32]. A comprehensive literature review on blood conservation tubes summarized multiple studies assessing cfDNA stability by comparing EDTA to Streck (*n* = 22); EDTA to PAX (*n* = 6); and one multi-tube comparison including EDTA, Streck, PAX, and Norgen. When processed within 72 h, no major differences in cfDNA stabilization efficacy were observed between the investigated stabilization tubes [33]. Concordantly, we found no significant difference in cfDNA and total DNA yields between the different cell-stabilization tubes, and only minor differences in cfDNA purity even after longer storage times of up to 7 days. When we then assessed the integrity of cfDNA by amplifiable genome equivalents, cfDNA from EDTA tubes was superior to cfDNA from blood preservation tubes decreasing from PAX over Norgen to Streck. In all preservation tubes, slightly less cfDNA was detectable after 7 days compared to 3 days. Therefore, processing is recommended at the earliest possible time point. These findings are in agreement with observations by other groups [34,35,36,37]. While EDTA tubes are not a realistic option for routine liquid biopsy analysis, especially in the context of multicenter trials with centralized sample processing, all tested conservation tubes can overcome the need for direct plasma generation.

Apart from studies investigating small RNAs for their use as biomarkers [38,39,40,41], liquid biopsy studies on cfRNA are lagging behind cfDNA-based approaches. One explanation might be the highly unstable nature of RNA in general due to exposure to a variety of RNases in body fluids [42]. Nevertheless, early studies indicated the great potential of disease-specific RNA transcripts in the prediction of metastatic spread in breast cancer patients [43] and in metastasis monitoring in pancreatic cancer [44]. Moreover, cfRNA harbors the potential of gene fusion calling that, due to patient-specific fusions, is more difficult to detect on the cfDNA level [45]. Especially targetable alterations such as ALK- or NTRK-fusions could be leveraged as liquid biomarkers for the prediction of therapy response and residual disease [46,47].

As demonstrated here, blood preservation tubes do not preserve cfRNA equally well. Our results show that cfRNA concentrations alone are not sufficient to predict the success rates of functional assays. While preservation of plasma in the different tubes did not affect the total amounts of isolated cfRNA, only directly processed EDTA tubes and PAX tubes allowed the successful amplification and detection of the oncogenic ETV6-NTRK3 fusion by ddPCR. The crosslinking approach of Streck tubes might negatively affect RNA integrity. Concerning the performance of Streck tubes, our results are in concordance with previous studies [33,36]. One study compared cfRNA stability from EDTA and Streck tubes and they concluded that the Streck tube should only be used for cfDNA analysis [37]. When Streck tubes were compared against five different blood preservation tubes in terms of microRNA yields and stability, Streck again showed the poorest results [36]. In contrast, another study suggested Streck tubes to be superior for the analysis of cfDNA and circulating tumor cells [48]. Besides the Cell-free DNA BCT tubes from Streck presented here, Streck offers an alternative solution with the RNA Complete BCT for Cell-free RNA tubes. Since we aimed to establish protocols for the combinatorial use of cfDNA and cfRNA, we did not include these tubes, but they should be taken into account for studies focusing solely on cfRNA.

### 3.2. cfNA Isolation Kits

In addition to cfNA purity, cfNA yield is a prerequisite for sensitive tumor detection by liquid biopsies. For most cfNA isolation kits, the isolation principle is either based on binding of nucleic acids to silica columns, differential binding to magnetic beads, or differential precipitation in organic chemicals. The methods vary in recovery efficiency, size discrimination, and robustness. We did not include magnetic bead-based isolation kits, which were shown to have poorer performance in comparison with manual isolation protocols [49]. The QB kit showed lowest yields and lowest purity, while QNA and QME revealed similar recovery efficiencies of cfDNA with minor contamination with larger fragments. Thus, we increased the cohort size for direct comparison of QNA and QME, which revealed QME’s superiority in terms of cfDNA yields while the purity was similar. Several studies have shown a good performance of the QNA kit, which made us confident that this was a good and reliable reference for the subsequent comparisons within our study [50,51,52,53]. For cfDNA applications, we found QME and NucleoSnap to show the most promising results concerning yield and purity. Although we only assessed NucleoSpin and NucleoSnap for their ability to co-isolate cfDNA and cfRNA, we found both of them to reliably isolate the cfNA they are intended to be used for, while also allowing co-isolation of the respective other cfNA to a satisfactory extent.

### 3.3. Quality Control for cfNA Analyses

For successful liquid biopsy analysis, two parameters should be routinely assessed. A fluorometric approach should be exploited for the estimation of total amount of isolated DNA and, subsequently, important information concerning size distribution should be assessed with electrophoresis, providing information about the degree of cellular contamination. One important consideration is that such extensive quality control should not consume significant amounts of material. For the fluorometric measurements, we showed an appreciable superiority of Qubit over PicoGreen. On a practical note, Qubit also allows faster processing, while PicoGreen requires more data points for the standard curve. The latter exists in both single-tube and 96-well plate-based formats, which is mostly beneficial for the quantification of higher numbers of samples. In contrast to a previous study, we did not observe different sensitivities for cfDNA and gDNA in PicoGreen measurements [54]. The electrophoretic approaches both showed accurate measurements of the expected cfDNA concentration and revealed informative size profiles and gDNA contamination. Concentrations below 100 pg/µL were still detectable in the BA profiles, and therefore electrophoretic methods allowed for quantification in ranges where no DNA was detected with comparable input volumes in the Qubit assay. On the basis of our experience, we recommend first obtaining fluorometric measurements with Qubit and subsequently complementing them with electrophoretic approaches, which together can help to predict and prevent experimental failure.

### 3.4. cfDNA Size Selection

In cancer patients, ctDNA fractions can be very low and may vary considerably. Still, the characteristic size distribution of cfDNA representing DNA protected from single, double, or triple nucleosomes can be used to enrich potentially tumor-derived material [28]. Some downstream applications, such as ligation-based library preparation protocols, preferably ligate short fragments and consequently do not require enrichment of shorter fragments beforehand. Similarly, many targeted capture approaches with short oligonucleotide baits bind short cfDNA more efficiently and thereby reduce the gDNA pool in the final library. Nevertheless, for quantification methods, gDNA contamination has to be taken into account and the sample input has to be adjusted. Too much gDNA can bias liquid biopsy analyses towards wild-type alleles when downstream applications, such as ddPCR, tagmentation-based methods, or chip-based methods, are applied. All these methods do not themselves exclude gDNA and therefore might require ultra-deep sequencing to exclude false-negative results. The latter methods thus benefit to a large extent from an in vitro size selection-based enrichment of the cfDNA. We here described a magnetic bead-based size selection protocol to enrich cfDNA fractions of one to three nucleosomes. The implementation of this protocol is feasible in every standard liquid biopsy lab. More sophisticated methods, such as microfluidic devices to separate cfDNA fractions from agarose cassettes, allow even sharper discrimination. Overall, irrespective of the chosen method, in vitro size selection increased a CNV-based score on lcWGS data of more than twofold and the single nucleotide variant score of two- to fourfold [28]. In vitro selection outperformed the in silico-based size selection in all tests and thereby proved the importance of an efficient size selection protocol [28].

### 3.5. Functional Readout

Most sequencing approaches depend on PCR amplification, which can be assessed by highly sensitive ddPCR assays to estimate the integrity of the isolated cfDNA and cfRNA fragments. For cfDNA, we employed the repetitive sequence of RPP30 to define the baseline of genome equivalent isolated. Interpreting the total amounts of cfRNA isolated from EDTA or blood preservation tubes alone was not informative in terms of cfRNA origin and cannot exclude unspecific RNA increase over time. However, targeted amplification of fusion transcripts ensures tumor specificity and should therefore not be affected by leukocyte lysis. To present the feasibility of cfRNA analysis in the tumor scenario, we showed the successful amplification of the oncogenic fusion ETV6-NTRK3, which might present a promising biomarker to monitor treatments with NTRK inhibitors. In addition, GAPDH transcripts were measured as transcript baseline control. The comparison of cfDNA methylation patterns revealed cfDNA methylation as a robust liquid biopsy read-out independent of the preservation tube used. This was assessed by tSNE clustering of the 10,000 most variable CpGs. This observation was consistent with a previous report on reduced representation bisulfite sequencing on cfDNA from a different set of preservation tubes [55].

### 3.6. Limitations of the Study

Our analysis was limited to three commercially available blood preservation tubes. Variables such as sample shipment and extreme temperature variations were not addressed in this study, but it was shown before that only extreme temperatures affected the tube performance [56]. One of the main advantages of liquid biopsies is the minimally invasive nature, which does not apply to the same extent to the more invasive nature of CSF sampling. However, in neurooncological patients, it has been shown to be an informative source for liquid biopsies. Due to comparatively low CSF sample volumes and very limited healthy reference material, CSF preservation for liquid biopsy studies has not yet been addressed. Within these limitations, we were able to directly compare preservation tubes for CSF only in fewer individuals than for blood plasma. However, we consider the results of the study as an important first step for the design of prospective brain tumor liquid biopsy studies. We used five different cfDNA or cfNA extraction kits, while cfRNA was only investigated for NucleoSpin and NucleoSnap. Our effective sequencing results are limited because that was not the focus of the study. In terms of ctRNA, we showed feasibility of detection even after 7 days, but we did not show data on ctRNA in real patients. Finally, further research remains essential to evaluate whether blood preservation tubes are compatible with methylation and deeper RNA analysis.

## 4. Material and Methods

### 4.1. Blood Preservation Tubes

#### 4.1.1. Plasma Isolation

For the direct comparison of commercially available cell-stabilizing tubes, we collected peripheral whole blood from four healthy individuals aged between 24 and 48 years by standard venipuncture using a Safety-Multifly needle 21G (Sarstedt, Nümbrecht, Germany). Informed consent was obtained from each participant, with protocols approved by the institutional ethics committee. Blood was drawn directly into 9 mL K_3_EDTA S-monovettes^®^ (Sarstedt, Nümbrecht, Germany) without cell-stabilizing additives and into cell-stabilizing tubes, namely, cf-DNA/cf-RNA Preservative Tubes from Norgen (Norgen Biotek, Thorold, ON, Canada), PAXgene Blood ccfDNA Tube (CE-IVD, PreAnalytiX, Qiagen, Hilden, Germany), and cell-free DNA BCT tubes (Streck, La Vista, NE, USA).

All tubes were filled up completely until the vacuum was exhausted. The tubes were inverted immediately after blood collection according to manufacturers’ recommendations. EDTA monovettes were centrifuged at 1900× *g* within 1 h of blood collection. Blood drawn directly into cell-stabilizing tubes was left at RT for 3 and 7 d. Adapting protocols to the specific requirements of liquid biopsies on the basis of experimental comparisons or communication with collaboration partners or manufacturers, we established and optimized standard operating procedures (SOP, cf. Protocol A1) to provide the best possible pre-analytical basis for high-quality plasma samples. Plasma isolation was performed according to the standardized protocols summarized in Table 1.

Each plasma supernatant was aliquoted into 2 mL Eppendorf tubes (Eppendorf, Hamburg, Germany) and immediately stored at −80 °C until further use. The collection was designed in a way that each sample had a matched EDTA sample that was processed immediately. Therefore, data collected from different individuals could be grouped to evaluate the tube conservation effect (Table S1).

#### 4.1.2. Cerebrospinal Fluid (CSF)

To investigate the utility of cell-stabilizing tubes for CSF collection, we collected CSF intra-operatively from a brain tumor patient and a non-oncological patient who provided informed consent to participate. The CSF was equally split into 1.5 mL aliquots and distributed into duplicates of Norgen (Norgen Biotek, Thorold, ON, Canada), PAX (PreAnalytiX, Qiagen, Hilden, Germany), and Streck (Streck, La Vista, NE, USA) tubes. Unconserved CSF was aliquoted into a 2 mL Eppendorf tube (Eppendorf, Hamburg, Germany). CSF was stored for 7 d at RT for blood preservation tubes and at 4 °C without conservative agent. Before centrifugation, one of each preservation tubes was filled up with phosphate-buffered saline (PBS, Thermo Fisher Scientific, Waltham, MA, USA), as recommended by Norgen. Centrifugation was performed according to Table 1 (SOP, cf. Protocol A2).

#### 4.1.3. Cell Culture Supernatant

In order to quantify ETV6-NTRK3 fusion transcripts in EDTA, Norgen, PAX, and Streck tubes, we collected cell culture supernatant from an ETV6-NTRK3 fusion-driven tumor cell line into the different tubes and processed it within one hour (EDTA tube) or kept it for 7 d at RT for the cell-stabilizing tubes. The cell line was established from tumor cells from a patient with inflammatory myofibroblastic tumor, and the fusion-transcript was confirmed by RNA sequencing analysis. After centrifugation, cfRNA was isolated with NucleoSpin, and fusion transcripts were assessed with ddPCR.

### 4.2. Isolation Kits

The isolation protocol and specific characteristics of all tested isolation kits are summarized in Table S2.

#### 4.2.1. cfDNA Isolation Kits

For the comparison of cfDNA isolation kits, plasma from 36 cancer patients (mean age 14.9 years, range 1–59 years) who provided informed consent to participate was processed with 2 or 3 different kits for pairwise comparison. Samples were thawed on ice before applying the different isolation kits. All centrifugation steps were performed at RT. DNA was always eluted in nuclease-free water (nf-H_2_O, Thermo Fisher Scientific, Waltham MA, USA) and stored at −80 °C until further analysis.

QIAamp DNA Blood Mini Kit (Qiagen, Hilden, Germany) was used following the instructions of the kit with the following modifications: Instead of 200 µL input volume, 600 µL plasma was isolated on one column. Proteinase K, Buffer AL, and 100% ethanol were adjusted to the appropriate ratio. Precipitated plasma lysate solution was centrifuged in several cycles through the membrane. The isolated DNA was eluted in 60 µL, and the eluate was passed twice over the membrane to maximize the yield. QIAamp Circulating Nucleic Acid Kit (Qiagen, Hilden, Germany) was used according to the manufacturer’s manual, with the exception that the isolated DNA was eluted in 35 µL and the eluate was applied on the column a second time to maximize the yield. QIAamp MinElute ccfDNA Mini Kit (Qiagen, Hilden, Germany) was used according to the manufacturer’s protocol, except that the isolated DNA eluted in 35 µL was run over the column a second time as well. For the NucleoSnap cfDNA kit (Macherey-Nagel, Düren, Germany), the manufacturer’s protocol was followed with a final elution volume of 50 µL.

#### 4.2.2. Combined cfNA Isolation Kits

Due to NucleoSnap cfDNA kit’s (Macherey-Nagel, Düren, Germany) superior performance in the first comparative experiments, we directly compared NucleoSnap to the NucleoSpin miRNA Plasma kit (Macherey-Nagel, Düren, Germany) concerning cfDNA yield and purity. Moreover, we evaluated cfRNA yields from both kits. For NucleoSpin, all samples were centrifuged at 4500× *g* before the start of the protocol. Skipping the optional DNA digestion step allowed for the parallel extraction of cfDNA and cfRNA. The final elution volume was 50 µL nuclease-free water (nf-H_2_O; Thermo Fisher Scientific, Waltham, MA, USA) after 5 min incubation on the membrane. Co-isolated cfNA was stored at −80 °C until further analysis.

### 4.3. Quantification

To exclude the effects of template fragment size, we utilized dilution series of short DNA fragments, cfDNA from cell culture supernatant (BT165) [57], and pooled gDNA for the comparative analysis of quantification methods.

All kits and devices were used as recommended by the manufacturers’ protocols. Reproducible quantification measurements were tested by applying a dilution series for cfDNA and gDNA with 11 different dilution steps. Fluorometric quantification was performed using technical replicates (*n* = 5). A total of 1 µL of DNA extracted from liquid biopsy samples was measured using the Qubit dsDNA HS Assay Kit (Thermo Fisher Scientific, Waltham, MA, USA) on the Qubit 3.0 Fluorometer (Thermo Fisher Scientific, Waltham, MA, USA). The detection range of the Qubit dsDNA HS Assay Kit (Thermo Fisher Scientific, Waltham, MA, USA) is between 0.5 and 500 ng/mL, with 100 pg total DNA being the lower detection limit for 200 µL/tube [58]. Measurement with Quant-iT PicoGreen dsDNA Assay Kit (Thermo Fisher Scientific, Waltham, MA, USA) was performed with 1 µL sample volume. Since the expected values were mostly in the range between 0 and 30 ng/µL, an adjusted, shortened standard curve from 25 pg/well to 30 ng/well was measured. Another standard curve was generated for values between 25 and 10 ng/well. The detection range of the Quant-iT PicoGreen dsDNA Assay Kit is between 0.25 ng/mL and 1000 ng/mL, with 50 pg being the lower detection limit for 200 µL/well [59]. The Bioanalyzer High-Sensitivity DNA Kit (Agilent, Santa Clara, CA, USA) was used according to manufacturer’s instructions using 1 µL per sample. The 7 lowest of the dilution steps were performed and quantified. The TapeStation Cell-free DNA ScreenTape system (Agilent Technologies, Santa Clara, CA, USA) was used with an input of 2 µL per sample for the 8 lowest dilution steps. Due to the upper limit of DNA input for BA and TapeStation, the higher concentrations were excluded. The different quantification protocols and specific characteristics of all tested quantification kits are summarized in Table S3 (SOP, cf. Protocol A3).

### 4.4. Size Selection

DNA samples that showed a high degree of gDNA contamination in BA profiles were subjected to size selection with AMPure XP beads (Beckman Coulter, Brea, CA, USA). Size selection was performed as previously described [60,61]. AMPure XP beads (Beckman Coulter, Brea, CA, USA) were added to the sample in a 0.6× ratio, incubated for 5 min at RT, and then put onto a magnet for 3 min. The supernatant was transferred into a new 1.5 mL Eppendorf tube (Eppendorf, Hamburg, Germany) and new beads were added to a 2.0× ratio. Samples were incubated for 5 min at RT and put onto a magnetic rack for 3 min. The supernatant was discarded. The beads were washed with fresh 80% ethanol (VWR International, Radnor, PA, USA) three times, followed by elution in 30 µL nuclease-free water (nf-H_2_O, Thermo Fisher Scientific, Waltham, MA, USA). After 5 min incubation time at RT, the tubes were transferred to a magnetic rack and incubated for 3 min. The supernatant was transferred into a fresh tube (2.0× sample). Samples were stored at −80 °C until further processing. (SOP, cf. Protocol A4).

### 4.5. ddPCR

Genome and transcriptome representation and integrity of cfDNA and cfRNA were analyzed by respective ddPCR assays. The cfDNA assay targets a 67 bp amplicon in RPP30 (Bio-Rad, Hercules, CA, USA), a non-amplified region in the human genome, to estimate cfDNA levels. On the transcriptome level, we designed an ETV6-NTRK3 fusion assay with a 75 bp amplicon and a GAPDH assay targeting an intron-spanning 123 bp amplicon. Primers (Sigma Aldrich, St Louis, MO, USA) and probes (Integrated DNA Technologies, Coralville, IA, USA) were designed using the Primer3Plus software (https://primer3plus.com/, assessed on 31 July 2020). To increase the signal-to-noise ratio, we used two quenchers for the ETV6-NTRK3 and GAPDH probes. ddPCR reagents were set up using the QX200 Droplet Digital PCR System (Bio-Rad, Hercules, CA, USA) as follows. For DNA detection, each reaction was set up in 20 µL reaction volume containing 10 µL 2× Supermix for Probes (no dUTP) and 2 µL 20× primer–probe mix, resulting in primer and probe concentrations of 900 nM and 500 nM, respectively, as well as 6–9 µL of input DNA.

For RNA detection, the One-Step RT-ddPCR protocol was used. A total of 20 µL reaction volume contained 5 µL of Supermix, 2 µL reverse transcriptase, 1 µL 300 mM DTT, 900 nM primer, and 500 nM probe, along with 6–9 µL of input RNA. Droplet generation was performed following the manufacturer’s manual using the QX200 Droplet Generator (Bio-Rad, Hercules, CA, USA). Thermal cycling was performed on a C1000 Touch Thermal Cycler (Bio-Rad, Hercules, CA, USA) as described in Table 2.

After thermal cycling, plates were left at 4 °C for at least 30 min to maximize droplet number readout [62]. Fluorescence intensity of probes was measured on a QX200 Droplet Reader (Bio-Rad, Hercules, CA, USA) and analyzed using QuantaSoft software version 1.0.596 (Bio-Rad, Hercules, CA, USA). Wells containing more than 10,000 droplets were assessed, and thresholds were chosen for each ddPCR assay on the basis of positive and no-template controls. The levels of cfNA were calculated as copies per well, and Poisson statistics were applied to convert the number of positive droplets to estimated targets, assuming independent segregation of molecules into the droplet reaction chambers. The total number of amplifiable cfNA per tube (copies/tube) was normalized for elution volume and plasma input. The assay setup is detailed in Table 2.

### 4.6. Methylation Analysis by EPIC-Array

Genome-wide DNA methylation profiles were obtained for the tumor reference and plasma samples (tube comparison, EDTA, PAX, Norgen, and Streck) using the Infinium MethylationEPIC (850k) BeadChip (Illumina, San Diego, CA, USA) [63]. Raw data were generated at the Genomics and Proteomics Core Facility of the German Cancer Research Center (DKFZ). The conumee package (Bioconductor) was used to infer genome-wide CNVs [64]. cfDNA methylation profiles resulting from preservation in the described tubes were analyzed in R. tSNE clustering was conducted on the M-values of the 10,000 most variable CpGs using the Rtsne and Rspectra packages.

### 4.7. lcWGS

lcWGS libraries were prepared with Accel-NGS 2S Hyb DNA Library Kit (Swift Biosciences, Ann Arbor, MI, USA) according to manufacturer’s protocol. cfDNA input was adjusted to 100 pg per library quantified by BA taking into account the cfDNA peaks (160–500 bp). Libraries were pooled and sequenced on a NovaSeq 6000 (Illumina, San Diego, CA, USA) to generate 100 bp paired-end reads.

### 4.8. Copy-Number Profiling

The paired-end reads data were transferred to DFKZ Omics IT and Data Management Core Facility (ODCF) for downstream analysis. Standard pre-processing analysis were processed by ODCF in-house AlignmentAndQCWorkflows (v1.2.73–1). Briefly, the workflow includes sequence alignment onto human reference genome GRCh37, marking duplicate reads, sorting, and extracting sequence quality matrix. CNV calling, segmentation, and tumor fraction estimating tool ichorCNA (v0.3.2) were deployed to calculate normalized log_2_ ratios from read count information for each genomic window of size 1 MB. Comparison and visualization of CNV profiles from matching tumor and plasma were performed in R (v3.6.0) environment.

### 4.9. Statistical Analysis

Data management and calculations were performed using GraphPad Prism 8 (Graphpad, Software Inc., La Jolla, CA, USA). Comparisons between two groups were performed using the Wilcoxon matched-pairs signed-rank test and unpaired *t*-test. For comparison of more than two groups, Friedman test or Kruskal–Wallis test, followed by Dunn’s multiple comparisons test, was performed. Statistical significance for the performance of blood preservation tubes was always tested against EDTA. *p*-values of <0.05 were considered statistically significant, with *p*-values represented as follows: * *p* < 0.05, ** *p* < 0.01, *** *p* < 0.001, and **** *p* < 0.0001. “ns” denotes differences in means that were not significant. All error bars shown represent standard deviation if not stated otherwise.

## 5. Conclusions

The multitude of commercially available products related to liquid biopsies can complicate the choice of pre-analytical workflows, especially when being new to field. With this study, we aimed to assist people making a well-informed choice for a pre-analytical workflow that suits best their scientific question such as fusion detection on cfRNA level, sequencing of cfDNA, or array-based methylation analysis of the latter. This manuscript provides a basis also for new products on the market with allowing a twofold comparison to the herein described best performing protocols.

To provide some guidance from sampling to sequencing, we describe a comprehensive protocol for liquid biopsy researchers developed by unbiased testing and evaluation of different technologies (see Graphical Abstract). Our study shows that plasma from blood preservation tubes has the potential for a combined analysis of both cfDNA and cfRNA from the same plasma sample. The described cfNA isolation kits, cfNA quantification kits, and quality control are now routinely used in our lab to process liquid biopsy samples. We observed clear differences between plasma samples from different clinics and accounted for this with processing adaptations summarized in this study, thereby minimizing the effect of pre-analytical variability in cfNA analysis. The implementation of standardized sampling and pre-analytical processing will allow for a direct sample to sample comparison, e.g., monitoring tumor content over time as a measure of therapy response. Altogether, our data can assist diagnostic laboratories in the design of future liquid biopsy studies and ultimately open the path to the use of liquid biopsies in therapy monitoring and long-term follow-up of cancer patients.

## Figures and Tables

**Table 1:** Characteristics of different blood preservation tubes and plasma isolation parameters.

	cfNA Preservation	Volume	Storage Conditions		Additive	Centrifugation Conditions		
Norgen	DNA, RNA	8.4 mL	30 d at RT		Osmotic cell stabilization	20 min	500× *g*	RT
PAX	DNA, RNA	10 mL	14 d at RT	28 d at 4 °C	Biological apoptosis prevention	15 min	1900× *g*	RT
Streck	DNA	10 mL	14 d at RT		Chemical crosslinking	15 min	1600× *g*	RT
EDTA	DNA, RNA	9 mL	1 h at 4 °C		$K_{3}\mathrm{EDTA}$	10 min	1900× *g*	4 °C

**Table 2:** ddPCR conditions. Assay specifics for detection of RPP30 (Ribonuclease P/MRP Subunit P30), ETV6-NTRK3 (fusion of ETS Variant Transcription Factor 6 and Neurotrophic Receptor Tyrosine Kinase 3), and GAPDH (glyceraldehyde-3-phosphate dehydrogenase). Probes were labeled with FAM (5(6)-Carboxyfluorescein) or HEX (Hexachloro-fluorescein). For improved self-quenching, we used double probes for RNA detection. In addition to Iowa Black^®^ FQ (3IABkFQ/), an internal ZEN™ quencher, was used.

	DNA Assay		Fusion Assay		RNA Assay	
**Target**	RPP30		ETV6-NTRK3		GAPDH	
**Amplicon Size**	67 bp		75 bp		123 bp	
**Forward** **Primer** **Sequence**	RPP30 Copy Number Determination Assay (Bio-Rad)		5’CCTGAAGAGCACGCCAT’3		5’GGTGTGAACCATGAGAAGTATGA’3	
**Reverse** **Primer** **Sequence**			5’GCTTCAGCACGATGTCTCT’3		5’GAGTCCTTCCACGATACCAAAG’3	
**Probe** **Sequence**			5’56-FAM/TGCTGCACA/ZEN/ TCTGCTATTCTCCCA/3IABκFQ’3		5’HEX/AGATCATCA/ZEN/ GCAATGCCTCCTGCA/3IABκFQ’3	
**Cycling Conditions**	95 °C for 10 min		50 °C for 60 min		50 °C for 60 min	
			95 °C for 10 min		95 °C for 10 min	
	94 °C for 30 s	40 cycles	94 °C for 30 s	40 cycles	94 °C for 30 s	40 cycles
	60 °C for 1 min		54 °C for 1 min		54 °C for 1 min	
	98 °C for 10 min		98 °C for 10 min		98 °C for 10 min	
	4 °C ∞		4 °C ∞		4 °C ∞	

## Supplementary Materials

The following are available online at https://www.mdpi.com/article/10.3390/cancers13123002/s1. Table S1, Summary of the study cohort illustrating the direct comparison of tubes and isolation methods applied, Table S2, Comparison of cell-free DNA extraction kits highlighting similarities and differences, Table S3, Comparison of cfDNA quantification kits considering distinct technical aspects, Figure S1, Comparison of cfNA yields after isolation with NucleoSnap and NucleoSpin, Figure S2, Comparative analysis of blood conservation tubes, Figure S3, Strategies for CSF conservation in blood preservation tubes, Figure S4, Comparative evaluation of cell-free DNA kit performance on yield and purity, Figure S5, Size selection strategies for the specific enrichment of cell-free DNA, Figure S6, GAPDH detection Transcriptome equivalents from plasma from indicated tubes represented as GAPDH copies plotted as copies per tube. Error bars show standard deviation (n = 4), Protocol A1, Blood Collection in Cell-Stabilizing Blood Collection Tubes, Protocol A2, CSF Collection in Cell-Stabilizing Blood Collection Tubes, Protocol A3, Quantification and Quality Control after cfDNA Isolation, Protocol A4, Right Side Size Selection for cfDNA Enrichment.

## Abbreviations

BA	Bioanalyzer
bp	Base pairs
cfDNA	Cell-free DNA
cfNA	Cell-free nucleic acids
cfRNA	Cell-free RNA
CSF	Cerebrospinal fluid
CNV	Copy number variation
ctDNA	Circulating tumor DNA
ctRNA	Circulating tumor RNA
ddPCR	Droplet digital PCR
EDTA	K_3_EDTA S-monovettes
gDNA	Genomic DNA
lcWGS	Low-coverage whole-genome sequencing
Norgen	cf-DNA/cf-RNA Preservative Tubes Norgen
NucleoSnap	NucleoSnap cfDNA kit
NucleoSpin	NucleoSpin miRNA Plasma kit
PAX	PAXgene Blood ccfDNA Tube IVD
PBS	Phosphate-buffered saline
QB	QIAamp DNA Blood Mini Kit
QME	QIAamp MinElute ccfDNA Mini Kit
QNA	QIAamp Circulating Nucleic Acid Kit
RT	Room temperature
SOP	Standard operating procedure
Streck	Cell-Free DNA BCT Streck
tSNE	T-distributed stochastic neighbor embedding
