# Supplementary material for: From Sampling to Sequencing: A Liquid Biopsy Pre-Analytic Workflow to Maximize Multi-Layer Genomic Information from a Single Tube
Source: Cancers (Basel). 2021 Jun 15;13(12):3002. doi: 10.3390/cancers13123002 (PMC8232701; doi:10.3390/cancers13123002)
Supplement: Supplementary file 1 [file cancers-13-03002-s001.zip › cancers-1181776-supplementary.pdf]

Table S1. Summary of the study cohort illustrating the direct comparison of tubes and isolation methods applied**.** T1–T4—Plasma samples collected from healthy individuals in EDTA (red), Norgen (blue), PAX (purple), and Streck (yellow) tubes isolated with NucleoSnap (red) and NucleoSpin (orange) for comparison of tube performances. C1—CSF sample collected from brain tumor patient in EDTA (red), Norgen (blue), PAX (purple), and Streck (yellow) tubes isolated with NucleoSpin (orange). Stars indicate PBS filling before centrifugation. SC2—Supplementary CSF sample collected from a healthy person in EDTA (red), Norgen (blue), PAX (purple), and Streck (yellow) tubes isolated with NucleoSpin (orange) for comparison of tube performance. P1-P7—Comparison of QIAamp DNA Blood Mini Kit (QB, green), QIAamp Circulating Nucleic Acid Kit (QNA, light blue), and MinElute Kit (QME, dark blue) for cfDNA isolation. SP8-SP16—Supplementary plasma kit isolation comparison QNA (light blue) and QME (dark blue). P8-P36—Plasma kit isolation comparison QME (dark blue) and NucleoSnap (red).

	T1	T2	T3	T4	C1	C1	SC2	P1	P2	P3	P4	P5	P6	P7	SP8	SP9	SP10	SP11	SP12	SP13	SP14	SP15	SP16	P8	P9	P10	P11	P12	P13	P14	P15	P16	P17	P18	P19	P20	P21	P22	P23	P24	P25	P26	P27	P28	P29	P30	P31	P32	P33	P34	P35	P36
QB																																																				
QNA																																																				
QME																																																				
Nucleosnap																																																				
Nucleospin																																																					
EDTA						*																																														
Norgen						*																																														
PAX						*																																														
Streck						*																																														

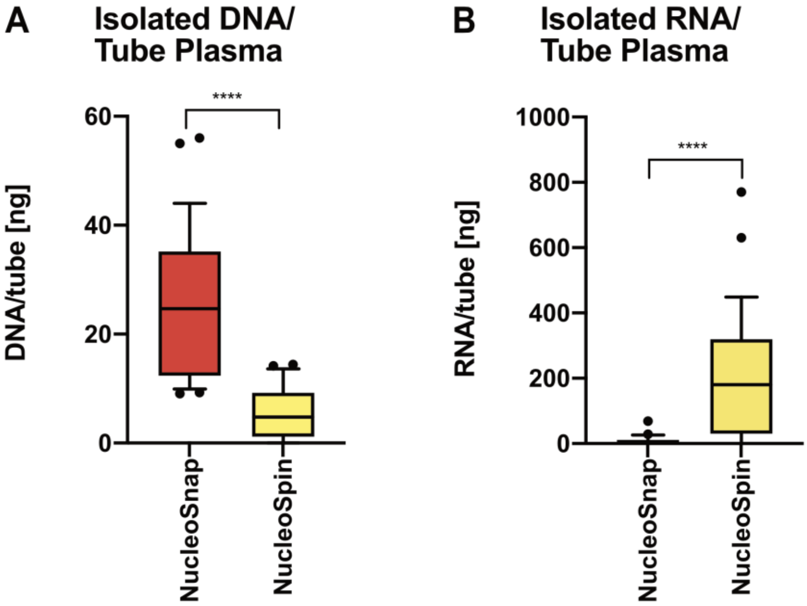

Figure S1. Comparison of cfNA yields after isolation with NucleoSnap and NucleoSpin. (**A**) DNA concentrations from NucleoSnap and NucleoSpin isolation. ****$p < 0.0001$ as determined by Mann Whitney U test. (**B**) RNA concentrations from NucleoSnap and NucleoSpin isolation. ****$p < 0.0001$ as determined by Mann Whitney U test.

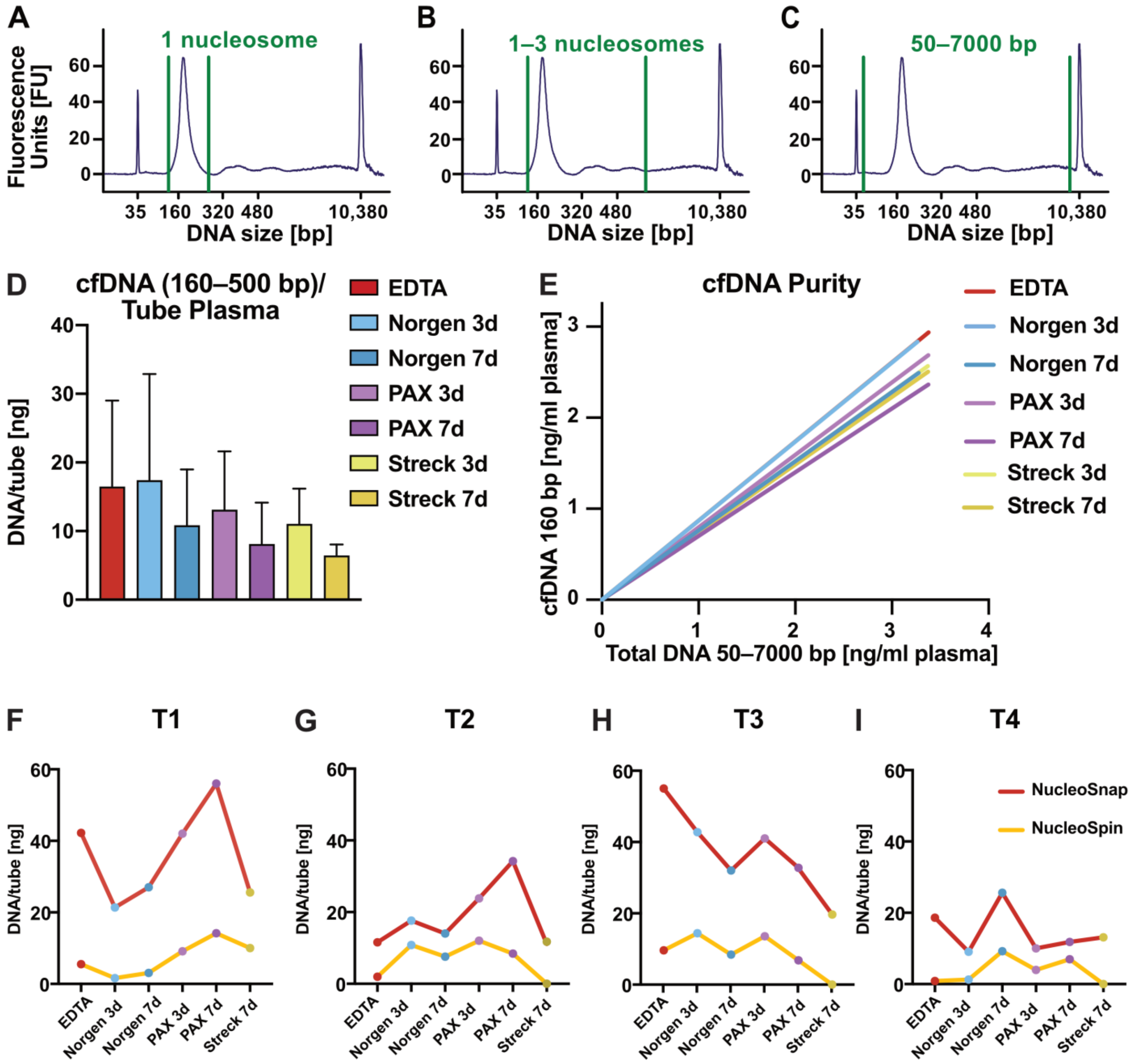

Figure S2. Comparative analysis of blood conservation tubes. (**A**) Representative Bioanalyzer (BA) profile with first nucleosomal (160 bp) region highlighted. (**B**) Representative BA profile with one to three nucleosome region highlighted. (**C**) Representative BA profile with region 50–7000 bp highlighted. (**D**) The mean concentration of cfDNA recovered from indicated tubes isolated by NucleoSnap quantified on Bioanalyzer profile (BA). The size of DNA wrapped around three nucleosomes is represented by 160 -500 bp ($n = 4$). (**E**) Direct comparison of cfDNA purity from EDTA, Norgen, PAX, and Streck tubes after isolation with NucleoSnap. Purity was defined as the ratio of cfDNA (first nucleosomal peak, 160 bp) to total DNA (50–7000 bp) measured by Bioanalyzer (BA). ETDA = 0.87; Norgen 3 d = 0.868; Norgen 7 d = 0.761; PAX 3d = 0.796; PAX 7d = 0.7; Streck 3 d = 0.762; Streck 7 d = 0.742. (**F**–**I)** Direct comparison of DNA yields from different tubes collected from the same person at the same time point isolated with NucleoSnap (red) or NucleoSpin (yellow) measured by Qubit for (**F**) donor 1 = T1, (**G**) donor 2 = T2, (**H**) donor 3 = T3, (**I**) donor 4 = T4.

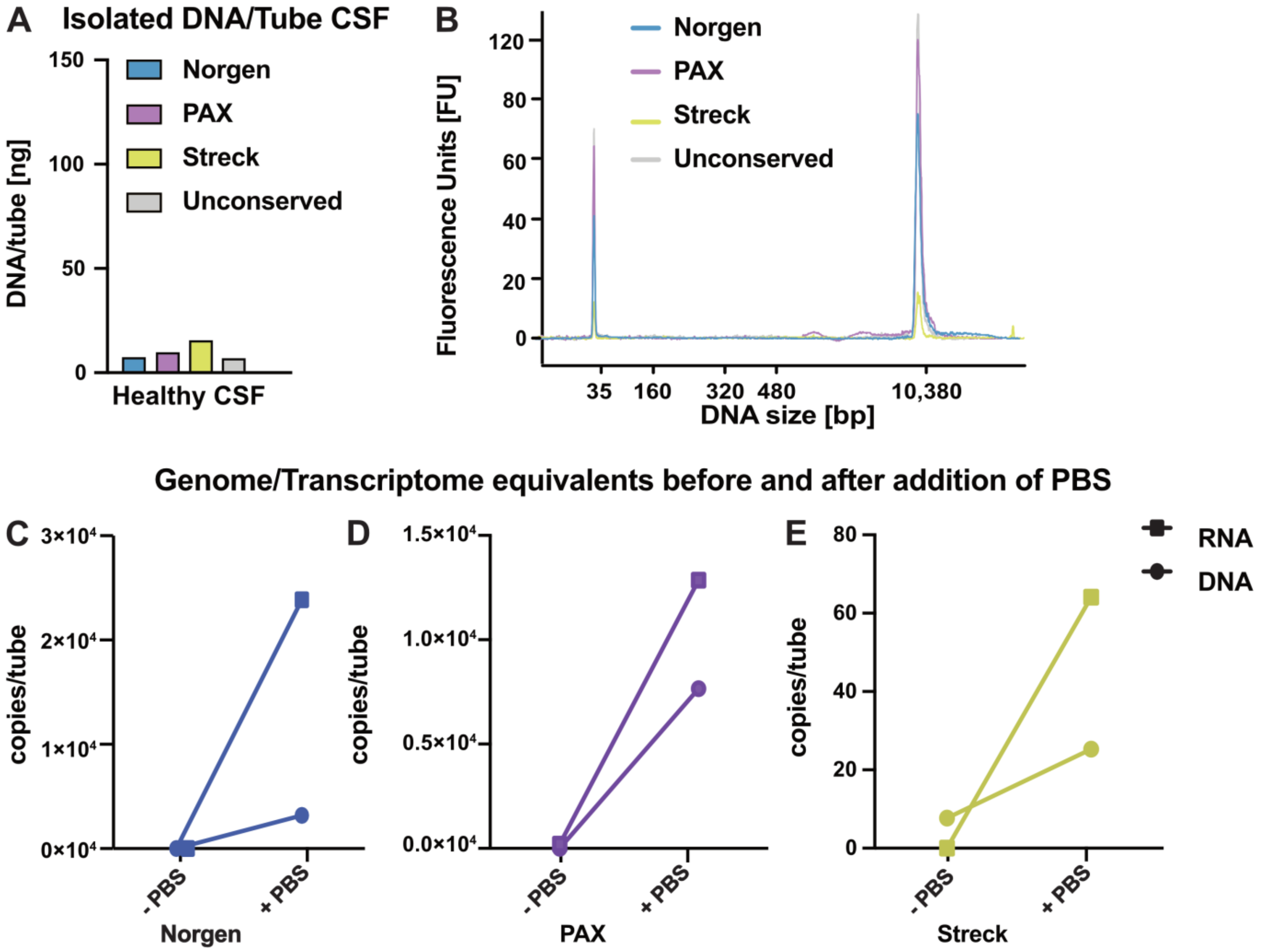

Figure S3. Strategies for CSF conservation in blood preservation tubes. (**A**) Concentration of total DNA recovered from 1.5 mL CSF of a non-oncological patient conserved in the indicated tube type. DNA was isolated with NucleoSpin, and the DNA concentration was measured by Qubit. (**B**) Bioanalyzer (BA) profiles for DNA isolated from CSF of a non-oncological patient after storage for 7d in a Norgen tube (light blue), a PAX tube (purple), a Streck tube (yellow), and unconserved (gray). (**C**–**E**) Genome and transcriptome equivalents from CSF in indicated tubes without (left) and with (right) the addition of PBS after CSF collection represented as copies per tube. Genome equivalents are represented by the non-amplified genome region RPP30 and transcriptome equivalent as copies of the housekeeping gene GAPDH per tube. (**C**) Norgen tube, (**D**) PAX tube, (**E**) Streck tube.

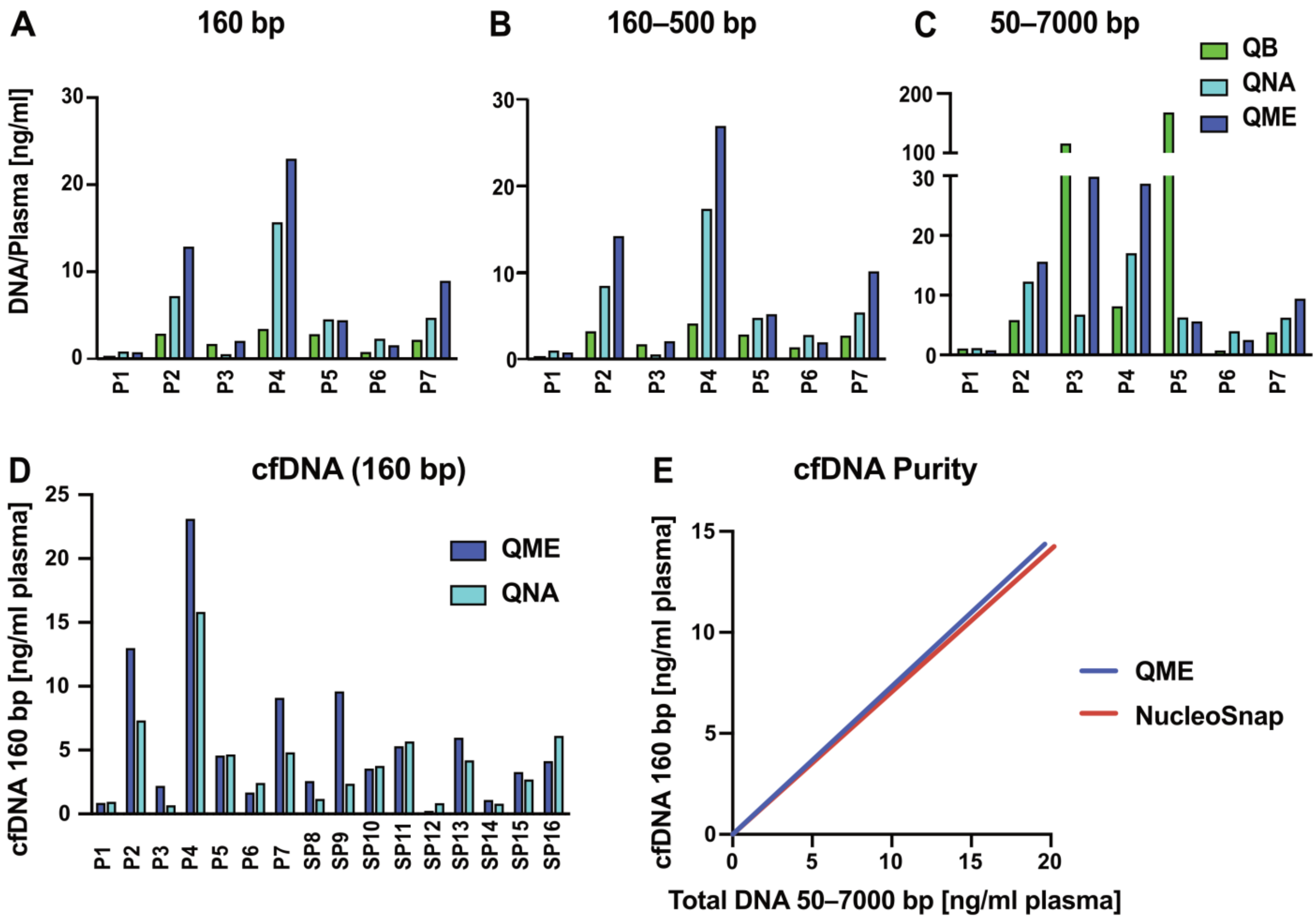

Figure S4. Comparative evaluation of cell-free DNA isolation kit performance on yield and purity. (**A**–**D**) Comparison of three purification kits: QIAGEN QIAamp DNA Blood (QB, green), QIAGEN QIAamp Circulating Nucleic Acid (QNA, light blue), and QIAGEN QIAamp MinElute ccfDNA (QME, dark blue). Plasma aliquots from the same collection time point from 7 pediatric cancer patients were subjected to the three kits. The amount of isolated DNA was standardized to 1 mL plasma for comparability. To compare yield and purity, we defined three DNA size ranges. (**A**) The concentration of DNA fragments with the length wrapped around one nucleosome and thereby protected from degradation ranging from around 146 to 176 bp and is represented by the 160 bp Bioanalyzer (BA) peak. (**B**) The concentration of DNA fragments corresponding to the length wrapped around one, two, and three nucleosomes including BA peaks of around 160–50 bp in DNA. (**C**) Quantification of total DNA including genomic contamination ranging from 50 to 7000 bp in size. (**D**) Pairwise comparison of QME and QNA applied to 16 patient plasma samples. cfDNA yields are depicted as the amount of cfDNA (160 bp). (**E**) Pairwise comparison of cfDNA purity after isolation with QME and NucleoSnap. Purity was defined as the ratio of cfDNA (first nucleosomal peak, 160 bp) to total DNA (DNA between 50–7000 bp) measured by BA. QME = 0.732; NucleoSnap = 0.706.

Table S2. Comparison of cfNA isolation kits with regard to distinct technical aspects.

DNA-Isolation-Kit	QIAamp DNA Blood Mini	QIAamp Circulating Nucleic Acid	QIAamp MinElute ccfDNA	NucleoSnap cfDNA	NucleoSpin miRNA Plasma
Technology	Silica membrane	Silica membrane	Preconcentration: Magnetic beads Silica membrane	Silica membrane	Silica membrane
DNA target size<br>RNA target size	20 - 30 kbp<br>no	< 1000 bp<br>< 1000 nt	< 1000 bp<br>no	50 - 1000 bp<br>50 - 1000 nt	50 - 1000 bp<br>15 - 1000 nt
Sample input	> 200 µl: DNA binding in several steps	Up to 5 ml	Mini 1-4 ml<br>Midi: 4-10 ml	1 - 10 ml	> 300 µl
Elution volume	50 - 200 µl	20 - 150 µl	20 - 80 µl	20 - 50 µl	20 - 50 µl
Preparation time	~40 min for 6 samples	< 2 h for 24 samples	1 h for 24 samples	1-2 h for 12 samples	1 h for 12 samples
Centrifugation before lysis	no	no	no	yes	no
Lysis	Proteinase K + Lysis Buffer	Proteinase K + Lysis Buffer	Proteinase K + Bead solution	Proteinase K + Lysis Buffer	Lysis Buffer
Pre-concentration	no	no	yes	no	no
DNA binding	spin	vacuum	spin	vacuum	spin
Membrane wash	2 x	2 x	1 x	2 x	3 x
Membrane dry	spin	spin	spin	spin	spin
Elute DNA	nuclease-free H_2O	nuclease-free H_2O	nuclease-free H_2O	nuclease-free H_2O	nuclease-free H_2O

Table S3. Comparison of cfDNA quantification kits considering distinct technical aspects.

	**Bioanalyzer High Sensitivity DNA Assay**	**TapeStation Cell-free DNA ScreenTape Assay**	**Qubit cfDNA High Sensitivity Kit**	**Quant-iT PicoGreen dsDNA Kit**
**Assay Principle**	Electrophoretic	Electrophoretic	Fluorometric	Fluorometric
**Analytic Device**	Chip	ScreenTape	QubitTubes	Microplate
**Sample Volume**	1 µl	2 µl	1–20 µl	1–20 µl
**Quantification Range**	5–500 pg/µl	20–4,000 pg/µl	50–100,000 pg/µl	2.5–100,000 pg/µl
**Detection Limit**	5 pg	40 pg	100 pg	50 pg
**Size Range**	50–7,000 bp	50–800 bp	No size information	No size information
**Analysis Time**	45 min/11 samples	1–2 min/sample	2 min/sample	10 min/96 samples<br>(incl. standard curve)

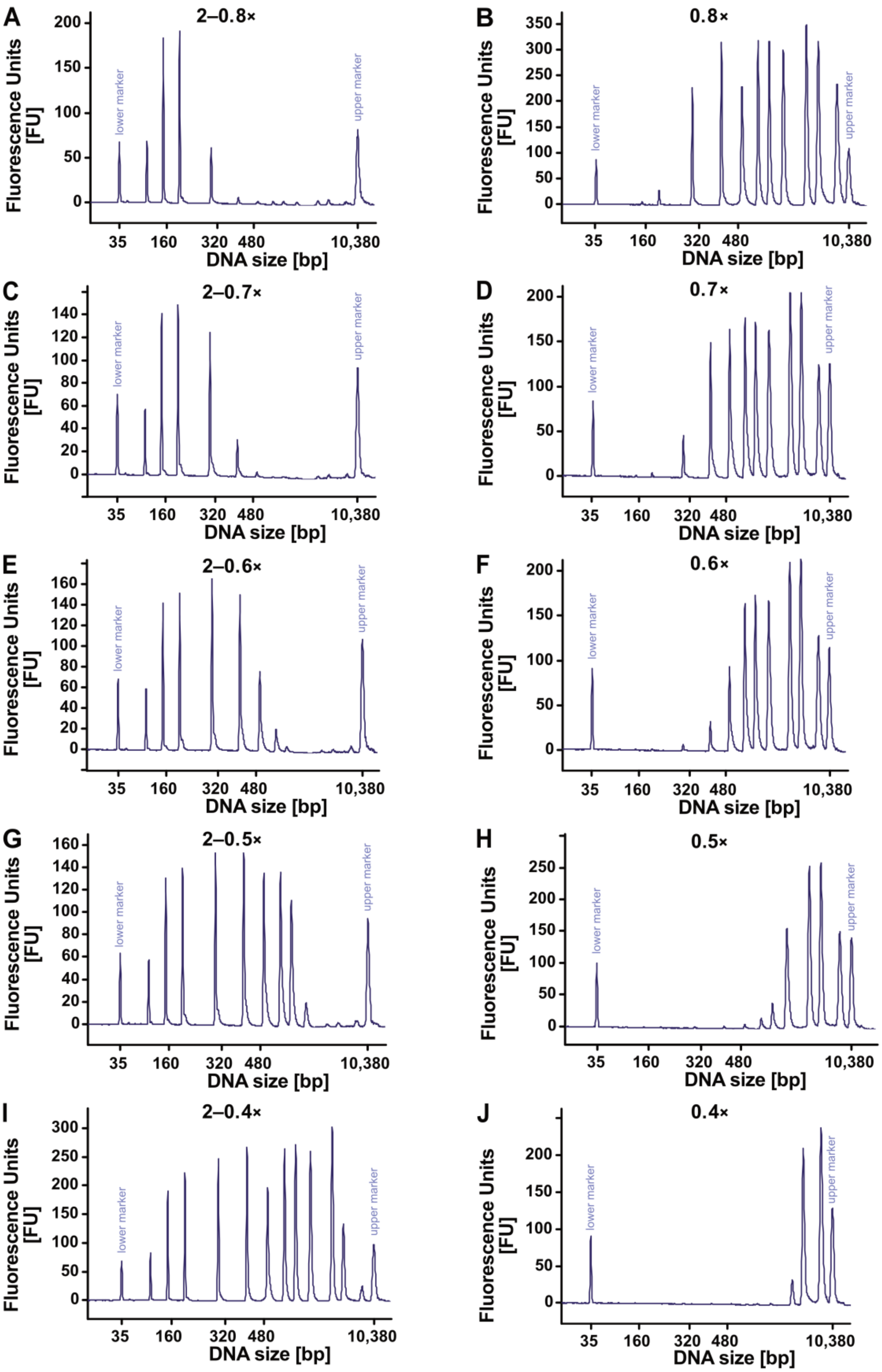

Figure S5. Size selection strategies for the specific enrichment of cell-free DNA. Representative Bioanalyzer (BA) profiles of the indicated Ampure bead ratio with supernatant fraction eluted with a 2.0x bead ratio on the left and respective bead fraction on the right. (**A**) Bead ratio 0.8, left side; (**B**) bead ratio 0.8, right side; (**C**) bead ratio 0.7, left side; (**D**) bead ratio 0.7, right side; (**E**) bead ratio 0.6, left side; (**F**) bead ratio 0.6, right side; (**G**) bead ratio 0.5, left side; (**H**) bead ratio 0.5, right side; (**I**) bead ratio 0.4, left side; (**J**) bead ratio 0.4, right side.

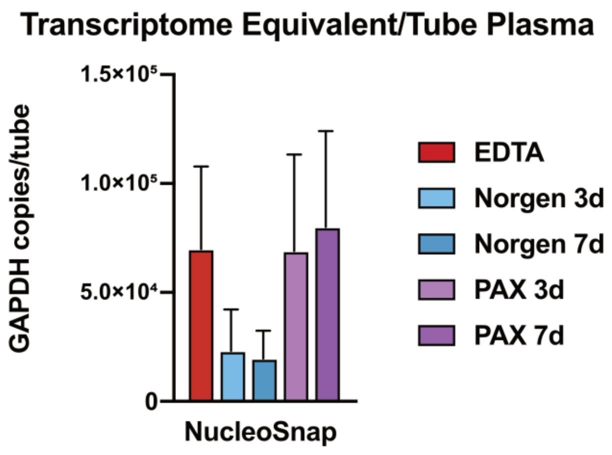

Figure S6. GAPDH detection transcriptome equivalents from plasma from indicated tubes represented as GAPDH copies plotted as copies per tube. Error bars show standard deviation (n = 4).

Appendix A—Workflow SOP

Protocol A1. Plasma collection in cell stabilizing blood collection tubes.
Protocol A2. CSF collection in cell stabilizing blood collection tubes.
Protocol A3. Quantification and quality control after cfDNA isolation.
Protocol A4. Right side size selection for cfDNA enrichment.

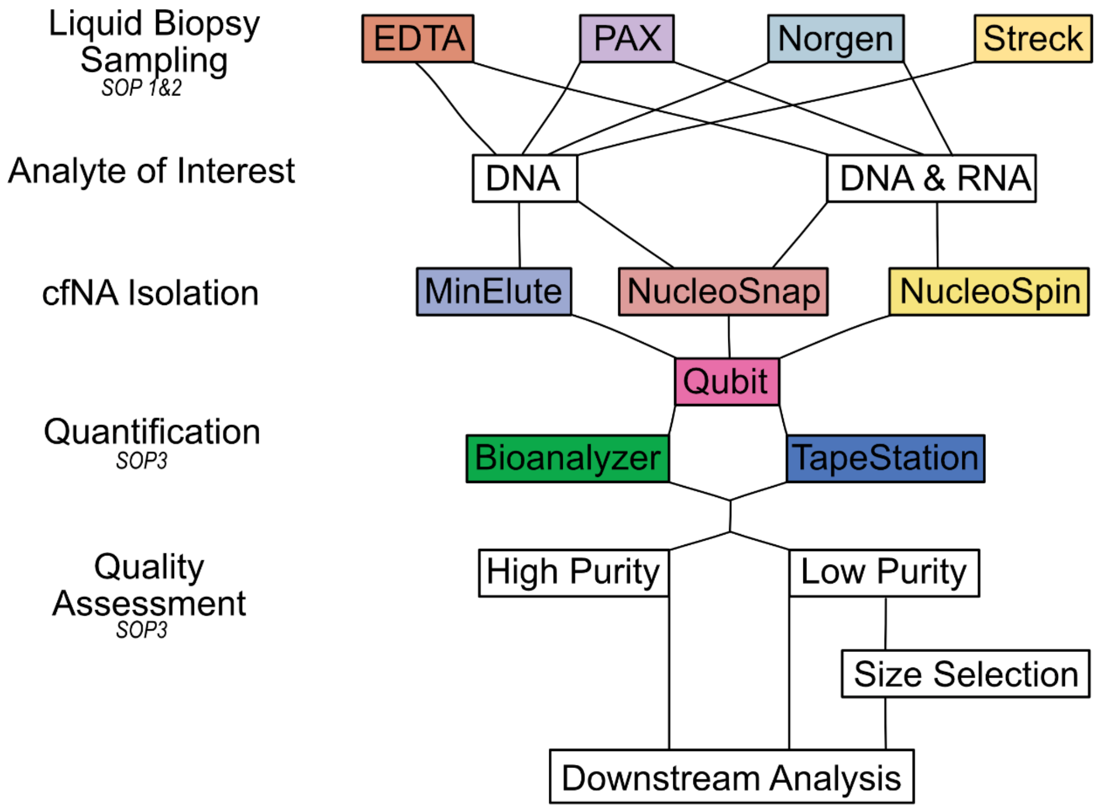

Protocol A1. Blood Collection in Cell-Stabilizing Blood Collection Tubes

I. **Material**

- Blood conservation tubes (BCT)
  - Norgen cfDNA/cfRNA preservative tubes (Norgen #63960)
  - or PAXgene Blood ccfDNA Tube (PreAnalytiX #768165)
  - or Cell-Free DNA BCT® Blood Collection Tube (Streck # 218997)
- Vacutainer Blood Collection Set or BD Vacutainer® blood transfer device
- Label (Patient-ID, collection date and collection time)
- Conical bottom centrifugation tube (e.g., Falcon 15 mL, #352096 or equivalent tube)

II. **Blood Collection in BCT**

1. Store new BCT at room temperature (15–25 °C)
2. Label tube with patient-ID, collection date, and collection time
   - Venipuncture with Vacutainer Blood Collection Set **or**
   - Collect blood with a sterile syringe and inject the blood with a cannula in the collection tube
   - Aim to completely fill the BCT
3. Invert each tube 8–10 times immediately after blood collection to ensure that the preservative makes uniform contact with the sample. This step is of critical importance!
4. Blood samples can be kept at room temperature (15–25 °C) for up to 30 days until further processing

III. **Procedure for Plasma Preparation with cfDNA/cfRNA Preservative Tubes**

Note: If less than 8.4 mL blood was collected, fill tube with 1x PBS (pH 7.4) up to blue cap before plasma preparation for cfDNA/cfRNA purification. DO NOT MIX and proceed with centrifugation.

Centrifuge blood samples depending on blood collection tube type:

1. Plasma processing through centrifugation
   a. **Norgen cfDNA/cfRNA preservative tubes**
   20 min at 500 × *g* at room temperature (15–25 °C)
   b. **PAXgene Blood ccfDNA Tube**
   15 min at 1900 × *g* at room temperature (15–25 °C)
   c. **Streck Cell-Free DNA BCT® Blood Tube**
   15 min at 1600 × *g* at room temperature (15–25 °C)
2. Transfer the plasma layer carefully into a new 15 mL conical bottom centrifugation tube (do not disturb the buffy coat and the cellular fraction, better to lose some microliters of plasma than take some cells from the buffy coat)

IV. **Plasma Storage**

1. For freezing, split collected plasma into aliquots of 1.8 mL in 2 mL Eppendorf tubes
2. Label plasma aliquots with patient-ID, collection day, collection time, and freezing time
3. Store plasma aliquots at −80 °C

Protocol A2. CSF Collection in Cell-Stabilizing Blood Collection Tubes

I. **Material**

- Norgen cfDNA/cfRNA preservative tubes (Norgen #63960)
- Cannula and syringe for CSF transfer into Norgen tubes
- Label (Patient-ID, collection date, and collection time)
- Conical bottom centrifugation tube (e.g., Falcon 15 mL, #352096, or equivalent tube)

II. **CSF Collection in Norgen cfDNA/cfRNA Preservative Tubes**

1. For liquid biopsy analysis, collect CSF in CSF collection tubes
2. Transfer CSF to Norgen tube either by injecting the CSF with a cannula or open the Norgen tube and add the CSF

3. Invert each tube 8–10 times immediately after CSF transfer to ensure that the preservative makes uniform contact with the sample. This step is of critical importance!
4. Blood samples can be kept at room temperature (15–25 °C) until further processing for up to 30 days

III. Procedure for CSF Preparation with Norgen cfDNA/cfRNA Preservative Tubes

Note: If the tube is not completely filled, add 1× PBS (pH 7.4) up to the blue cap before plasma/CSF preparation for cfDNA/cfRNA purification. DO NOT MIX and proceed with centrifugation.

Centrifuge CSF samples as follows:

1. Centrifuge Norgen cfDNA/cfRNA preservative tubes for 20 min at 500 × *g* at room temperature (15–25 °C)
2. Transfer the plasma or CSF layer carefully into a new 15 mL conical bottom centrifugation tube (do not disturb the buffy coat and the cellular fraction, better to lose some microliters of plasma than to take some cells from the buffy coat)

IV. Plasma and CSF Storage and Shipment

1. Label plasma and CSF samples with patient-ID, collection day, collection time, and freezing time
2. Store plasma and CSF samples at −80 °C until shipment
3. Ship plasma and CSF samples in batches

Protocol A3. Quantification and Quality Control after cfDNA Isolation

I. Equipment

- Qubit Fluorometer 3.0 (Thermofisher #Q33216)
- 2100 Bioanalyzer instrument (Agilent, #G2939BA)
- Chip priming station (Agilent, #5065-4401)
- Removable cartridge for DNA (Agilent, #5065-4413)
- IKA vortex mixer with attachment for Chip (MS 3 basic, #0003617000)

II. Material

- Qubit Assay Tubes (Thermofisher, #Q32856)
- Qubit dsDNA HS Assay Kit (Thermofisher, #Q32851)
- 0.2 mL PCR singleCap 8er stripes (Biozym, #710970)
- Bioanalyzer High-Sensitivity DNA Kit (Agilent, #5067-4626)

III. Software

- Agilent Bioanalyzer, 2100 Expert

IV. Qubit Fluorometer 3.0, dsDNA HS Assay

- Store DNA standards at 4 °C; before usage, adjust standards for 30 min to RT
- Set DNA input volume to 1 µL; if different volumes are measured, adjust the volumes in the fluorometer options accordingly
- Perform Qubit dsDNA HS-Assay Protocol according to manufacturer’s manual

V. Bioanalyzer HS DNA

1. Measurement with Agilent Bioanalyzer, High-Sensitivity DNA Kit
   - Store the Bioanalyzer HS DNA Kit at 4 °C; before usage, adjust reagents for 30 min to RT
   - Fill cleaning chip with 300µL nuclease-free water and clean electrodes before use
   - Dilute samples to a concentration of < 1.0 ng/µL
   - Perform assay according to Agilent High-Sensitivity DNA Kit Quick Start Guide
2. Analysis of HS DNA chip results with Agilent Bioanalyzer 2100 Expert Software
   - Measure the samples by adjusting the baseline correction
   - Control and potentially correct the lower and upper marker (position, width, integrated area)
   - Set first, second, and third peak by manual integration to assess cfDNA yields
   - Set region tables from 50 to 7000 bp to assess gDNA contamination

- Correct the reference points of the zero line for the region measurement

Protocol A4. Right Side Size Selection for cfDNA Enrichment

I. Equipment
- Vortexer
- Microcentrifuge (1.5–2 mL)
- DynaMag-2 Magnet (ThermoFisher, #12321D)

II. Material
- Eppendorf tubes 1.5 mL (e.g., Eppendorf #12682)
- Conical bottom centrifugation tube (e.g., Falcon 15 mL, #352096, or equivalent tube)
- Ampure Beads
  - **DNA**—Agencourt® AMPure® XP (Beckman #A63881) or
  - **RNA**RNAClean XP (Beckman #A63987)
  - Ethanol absolute (e.g., Merck # 107017)
  - Nuclease-free H_2O (e.g., Life Technologies, #AM9937)

III. AMPure Bead-based Size Selection
1. Store bead solutions at 4 °C; before usage, adjust bead solution for 30 min to RT
2. Prepare fresh 80% ethanol
3. Adjust sample volume with nuclease-free H_2O to 50 μL in a 1.5 mL Eppendorf tube
4. Add 30 μL Ampure beads to the sample (0.6× ratio)
5. Incubate for 5 min at RT
6. Transfer tube on magnetic rack and incubate for 3 min

→ 0.6× bead fraction contains large DNA fragments (proceed to step 19 to recover large fragments)

7. Transfer supernatant containing smaller fragments to a fresh 1.5 mL Eppendorf tube
8. Add the required number of beads to the supernatant:
   a. Sample volume (μL) × (2.0×—the initial ratio) = volume beads (μL)

   →50 μL × (2.0–0.6) = 70 μL of beads
9. Incubate for 5 min at RT
10. Put sample for 3 min on magnetic rack
11. Wash beads 3× for 30 s with 1000 μL 80% EtOH
12. After the last washing step, let the bead pellet dry for 5 min
13. Elute the smaller fragments in 30 μL nuclease-free H_2O
14. Resuspend beads rigorously by flicking the tube, quickly spin beads down
15. Incubate for 5 min at RT
16. Transfer tube on magnetic rack and incubate for 3 min
17. Transfer 30 μL eluate containing small cfDNA fragments into fresh tube

→0.6×–2.0× bead fraction contains small DNA fragments

18. Elute the larger fragments in 30 μL nuclease-free H_2O
19. Resuspend beads rigorously by flicking the tube, quickly spin beads down
20. Incubate for 5 min at RT
21. Transfer tube on magnetic rack and incubate for 3 min
22. Transfer 30 μL eluate containing small cfDNA fragments into fresh tube
